# Micro/Nano-Motors for Enhanced Tumor Diagnosis and Therapy

**DOI:** 10.3390/ijms26167684

**Published:** 2025-08-08

**Authors:** Zherui Zhang, Bulong Gao, Ruizhen Tian, Jiayun Xu, Tingting Wang, Tengfei Yan, Junqiu Liu

**Affiliations:** 1 College of Chemistry and Chemical Engineering, Central South University, Changsha 410083, China; zzr19951007@outlook.com; 2 Key Laboratory of Organosilicon Chemistry and Material Technology, Ministry of Education, College of Material, Chemistry and Chemical Engineering, Hangzhou Normal University, Hangzhou 311121, China; 2024112009077@stu.hznu.edu.cn (B.G.); tianrz17@163.com (R.T.); xujiayun@jlu.edu.cn (J.X.); tengfeiyan@163.com (T.Y.)

**Keywords:** micro/nano-motors, cancer diagnosis and therapy, synergistic therapy

## Abstract

Cancer remains one of the most significant diseases threatening human health. The lack of effective diagnostic and therapeutic technologies is a critical factor contributing to the high clinical mortality rates associated with malignant tumors. Self-propelled micro/nano-motors (MNMs) hold promise for addressing the limitations of conventional nanoparticles in cancer diagnosis and treatment. Their unique motion characteristics enhance the efficiency of MNMs in achieving rapid distribution, deep tissue penetration, and targeted delivery in vivo. This review systematically summarizes recent advances in MNM-based therapy for tumor diagnosis and treatment, offering a comprehensive overview of their material classification, self-propelled mechanisms, targeting strategies, and therapeutic approaches. Subsequently, we discuss the therapeutic mechanisms of MNMs within the tumor microenvironment in detail and highlight the advantages of synergistic multimodal therapies, including chemodynamic therapy, sonodynamic therapy, photothermal therapy, immunotherapy, photodynamic therapy, and gas therapy. Finally, we outline the main challenges and prospects for the development of MNMs in cancer diagnosis and therapy.

## 1. Introduction

Cancer remains one of the most significant public health challenges of the 21st century, responsible for approximately 30.3% of global premature deaths from noncommunicable diseases among individuals aged 30–69 years [1]. Despite remarkable advances in medicine and biology, cancer incidence and mortality rates continue to rise. Conventional oncology treatments—including surgery, radiotherapy, and chemotherapy—have achieved notable successes and technological improvements, yet they inevitably cause significant side effects [2,3,4,5,6,7,8,9]. These limitations include the frequent delay in early cancer detection, which results in missed opportunities for timely intervention [10]. Surgical resection alone often fails to ensure complete tumor eradication, thereby increasing the risks of recurrence and metastasis [11,12,13]. Prolonged use of chemotherapy drugs can lead to drug resistance in tumor cells and impose additional physical burdens on patients [14,15]. Targeted therapy requires precise identification of specific cancer cell mutations, limiting its wide applicability [16,17]. Furthermore, radiation therapy can induce significant damage to healthy tissues, resulting in considerable pain and suffering for patients [18]. Therefore, the development of precise early cancer diagnosis and effective tumor treatment strategies is crucial for cancer prevention and treatment.

With the rapid advancement of nanotechnology, nanomedicine offers unprecedented opportunities in both cancer treatment and diagnosis. It focuses on developing nanoscale materials that utilize nanotechnology to formulate drugs into nanoparticles, thereby enhancing their tumor accumulation efficiency [19]. These nanostructures can circulate in the bloodstream for extended periods, enabling precise and controlled drug release according to prescribed dosages [20]. Furthermore, as a nano-delivery platform, nanodrugs not only exhibit the intrinsic biological effects of nanomaterials but also facilitate the transport of diverse cancer therapeutic agents for combination therapy. Critically, they can leverage unique characteristics of the tumor microenvironment (TME) to achieve targeted drug release, thereby reducing the systemic side effects associated with conventional chemotherapy [6,21,22,23,24,25,26]. However, recent studies have identified several critical factors within the TME that impede nanoparticle accumulation in tumor tissue, including the dense extracellular matrix [27], solid stress [28], and abnormal vascular structures [29,30,31].

To achieve efficient cancer therapy, it is crucial to develop rationally designed delivery systems that can overcome multiple transport barriers encountered on the way to tumor sites. Micro/nano-motors (MNMs)—miniaturized devices capable of converting ambient energy into mechanical motion [32]—represent a promising strategy for addressing these challenges. offer promising solutions. Since the early 2000s, MNMs have received considerable attention in the fields of cancer diagnosis and therapy [33,34,35,36,37]. In biological systems, fuel-dependent MNMs typically utilize non-toxic or biocompatible fuels such as glucose, hydrogen peroxide (H_2_O_2_), or urea. These generate propulsion through chemical reactions (e.g., gas evolution) or concentration gradients [38,39,40]. For instance, Li et al. successfully synthesized JAuNR-Pt by depositing Pt nanoparticles at one end of a gold nanorod [41]. This structure effectively catalyzes the decomposition of H_2_O_2_, thereby generating a proton gradient that serves as a driving force. In addition, fuel-free MNMs, which are powered by various energy sources such as light [42,43,44], magnetic fields [45,46], and ultrasound [47,48,49], enable enhanced operational flexibility. These systems utilize energy-converting materials such as photothermal agents, magnetic composites, or piezoelectric substrates. For example, Wan et al. asymmetrically immobilized Pt nanoparticles on mesoporous–macroporous silica, where their photothermal effect propels nanomotors [50].

With the advancement of research, MNMs have expanded beyond nanomaterials. The unique tumor-targeting capability of microorganisms has increasingly drawn scientific interest, leading to the growing use of biological entities—including eukaryotic cells [51], platelet cell [52], bacteria [53], and viruses [54]—as drug delivery systems. Despite the recognition of bacteria’s anti-tumor effects as early as 1868, their intrinsic virulence and limited efficacy, coupled with the subsequent emergence of radiotherapy and chemotherapy, impeded progress in bacterial therapy [55]. A deeper understanding of TME and recent advances in genetic engineering have rekindled interest in bacterial carriers as potential next-generation cancer therapeutics. Hypoxic conditions within the TME promote the proliferation of anaerobic and facultative anaerobic bacteria [56,57,58], facilitating their spontaneous accumulation in tumor tissues. Furthermore, bacteria act as pathogens that activate the immune system, triggering autoimmune responses. This immunostimulatory effect within tumors also presents novel opportunities for immunotherapy (IT) [59]. For instance, Shi et al. genetically engineered a strictly anaerobic Salmonella strain (denoted △St). Combining △St with nano-catalytic therapy and photothermal therapy induced immunogenic cell death (ICD) in a substantial proportion of cancer cells [60]. In addition, natural cells also demonstrate inherent advantages—such as low immunogenicity, long lifespans, and strong binding specificity—making them ideal carriers for therapeutic and diagnostic agents [61,62]. Platelets exemplify these properties remarkably. Their considerable volume and surface area confer high drug-loading capacity. Coupled with their abundance, rapid turnover, and efficient targeting abilities, these characteristics establish platelets as an ideal platform for targeted drug delivery [63,64,65].

**Figure 1:**
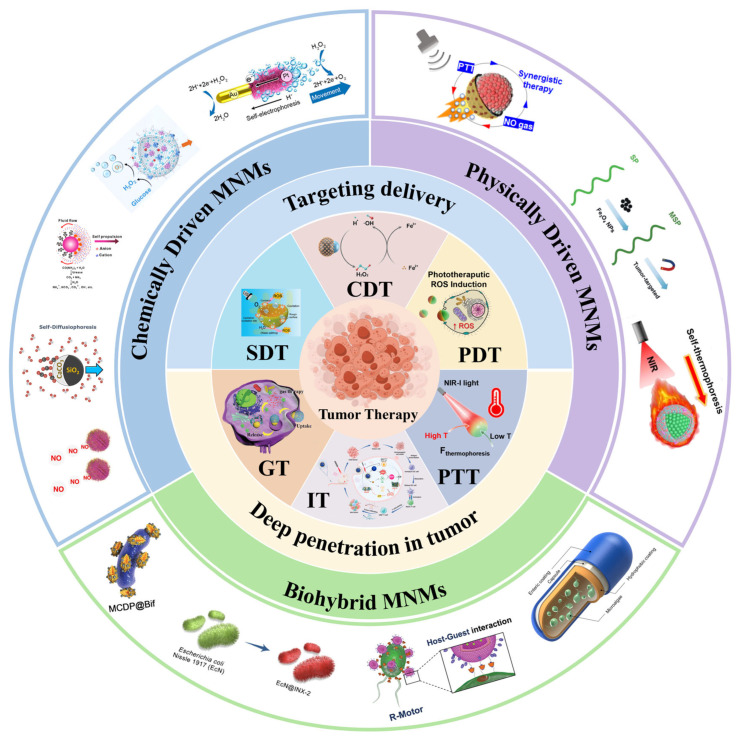
Schematic illustration of MNMs design, chemically driven, physically driven, and biohybrid strategies, showcasing their targeting, tumor penetration, and synergistic effects.

Comprehensive and detailed reviews already exist summarizing the development, preparation, propulsion mechanisms, and biological applications of MNMs [66,67,68]. While some of these include coverage related to cancer treatment [69], detailed reviews specifically on the synergistic treatment of cancer using MNMs combined with other therapies remain relatively scarce. To better advance the application of MNMs in cancer therapy, this review focuses on MNM-mediated therapeutic applications in cancer treatment, comprehensively examining their underlying mechanisms. We systematically introduce three key aspects: (1) movement mechanisms of MNMs for cancer therapy; (2) tumor-targeting and penetration capabilities of MNMs; (3) synergistic effects of combining MNMs with other therapies to enhance anti-tumor efficacy, including chemodynamic therapy (CDT), sonodynamic therapy (SDT), photothermal therapy (PTT), chemotherapy (CT), photodynamic therapy (PDT), immunotherapy (IT), and gas therapy (GT) (Figure 1).

## 2. Motion Mechanisms of MNMs

**Figure 2:**
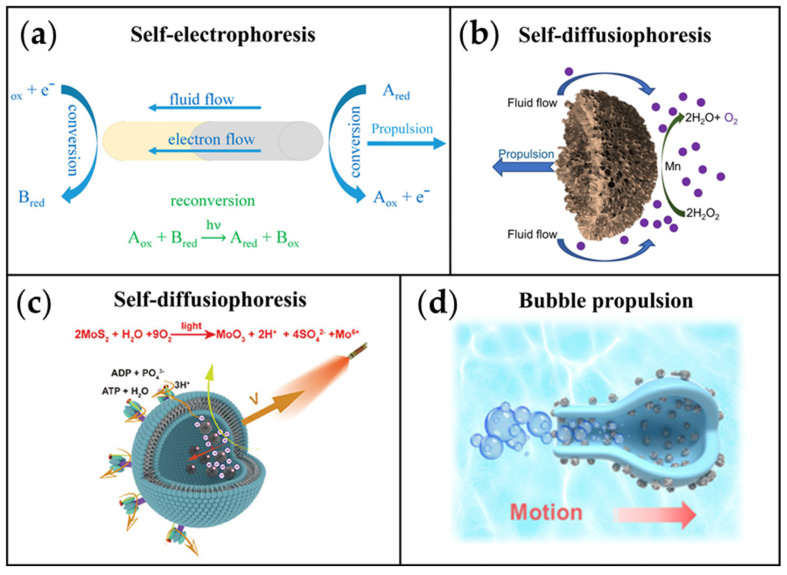
Schematic illustrations of chemically driven MNMs propulsion mechanisms: (**a**) Self-electrophoretic motion (reproduced from [74] with permission from the American Chemical Society). (**b**) H_2_O_2_-based self-diffusiophoretic propulsion (reproduced from [75] with permission from Elsevier). (**c**) Photo-triggered self-diffusiophoretic propulsion (reproduced from [76] with permission from John Wiley and Sons). (**d**) Bubble-propelled motion (reproduced from [77] with permission from the American Chemical Society).

The fundamental principle behind constructing MNMs involves harnessing energy from the surrounding microenvironment and converting it into propulsive force. To achieve this across diverse settings, researchers have developed various propulsion mechanisms. In this paper, we primarily categorize MNMs used in tumor therapy and those with anti-tumor potential using three criteria. The first criterion involves a classification based on the type of fuel consumed. Chemically driven MNMs convert chemical energy from environmental fuels into mechanical energy via surface or internal reactions, enabling autonomous motion [66,70]. The primary mechanisms by which chemical propulsion converts energy into motion are bubble propulsion [71], self-diffusion phoresis [72], and self-electrophoresis [73]. Self-electrophoretic MNMs utilizes asymmetric conductive materials where electrochemical half-reactions create an internal electron flow and external ion migration (e.g., protons), propelling the MNM opposite to the ion flux [74] (Figure 2a). Self-diffusiophoretic MNMs relies on catalyzing interfacial substrate transformations (e.g., via chemical [75] or photo-induced reactions [76]) to generate concentration gradients that induce fluid flow and motion to establish concentration gradients (Figure 2b,c). Bubble propulsion, particularly effective in complex biological environments and often outperforming the other mechanisms there, generates thrust from bubbles produced by catalytic or non-catalytic reactions(e.g., H_2_O_2_ decomposition: 2H_2_O_2_ → O_2_ ↑ + 2H_2_O) [77] (Figure 2d), expanding its repertoire beyond common O_2_ to include gases like NO [78] and H_2_S [79]. The second criterion is categorized according to the physical field required for MNMs’ movement. Physically driven MNMs harness external stimuli such as magnetic or electric fields, ultrasound, or light to power motion. In contrast to chemically propelled MNMs, these offer superior controllability over speed and direction, addressing challenges like the complex TME or insufficient endogenous fuel concentrations. However, this approach typically requires external equipment for precise control [80]. Extensive research has focused on light-, magnetic-, and ultrasound-driven MNMs for achieving targeted propulsion, which is critical for cancer therapy tasks like site-specific drug delivery. While chemical and physical drives represent the most widely adopted strategies for autonomous motion, biohybrid propulsion has emerged as a promising alternative in recent years [81]. Inspired by nature, whole biological entities such as bacteria, sperm, or algae act as bioengines [82,83,84]. These elements are integrated with synthetic materials to create biohybrid MNMs, capitalizing on the inherent selective affinity or chemotactic capabilities of the living systems to generate propulsive force.

### 2.1. Chemically Driven MNMs

#### 2.1.1. H_2_O_2_-Dependent MNMs

As a reactive oxygen species (ROS), H_2_O_2_ mediates diverse physiological functions, including cytokine signaling, insulin regulation, and intracellular redox homeostasis [85]. The generation of H_2_O_2_ in cells is closely associated with NADPH oxidase activity. In cancer cells characterized by high metabolic activity, the NADPH oxidase (NOX family) is frequently abnormally activated, leading to an abnormal increase in H_2_O_2_ levels within tumor cells [85]. Consequently, H_2_O_2_-dependent MNMs can effectively exploit this abundant substrate within the TME, a feature significant for tumor targeting, drug delivery, and penetration into tumor tissue.

**Figure 3:**
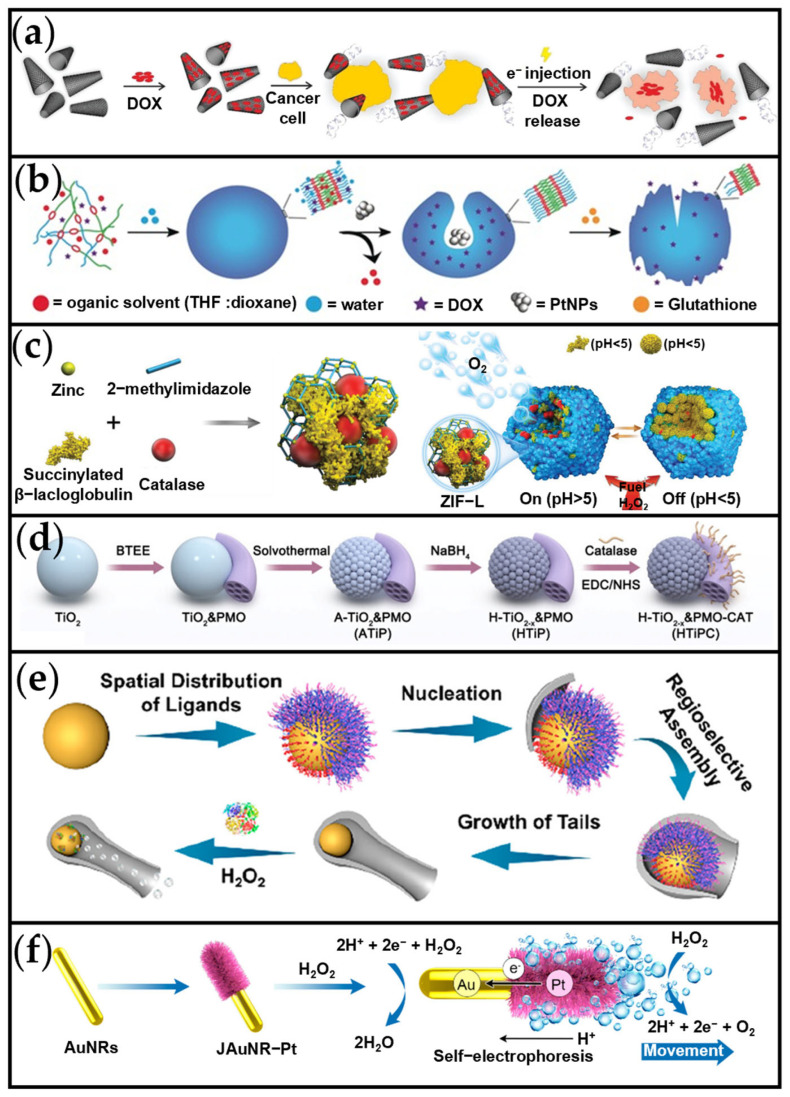
H_2_O_2_-dependent MNMs. (**a**) Schematic illustration of DOX loading and release on/from the n-rGO/Pt micromotors (reproduced from [88] with permission from John Wiley and Sons). (**b**) Self-assembly and GSH-triggered disassembly of the redox-sensitive stomatocyte nanomotor (reproduced from [89] with permission from John Wiley and Sons). (**c**) Schematic representation of micromotor assembly with pH-responsive on/off motion (reproduced from [90] with permission from John Wiley and Sons). (**d**) Schematic representation of HTiPC micromotor assembly (reproduced from [91] with permission from the American Chemical Society). (**e**) Schematic illustration of AHOAST superassembly and self-propulsion mechanisms (reproduced from [92] with permission from the American Chemical Society). (**f**) Schematic illustration of the synthesis pathway for the JAuNR-Pt nanomotor and the rapid self-propulsion of AuNRs toward the Pt side via self-electrophoresis (reproduced from [41] with permission from the American Chemical Society).

Ismagilov et al. developed a millimeter-scale self-propelled motor by integrating a polydimethylsiloxane (PDMS) plate with a Pt-plated porous glass [86]. This design utilized the recoil force generated by O_2_ bubbles (via the reaction 2H_2_O_2_ → O_2_ ↑ + 2H_2_O) during Pt-catalyzed H_2_O_2_ decomposition for propulsion. Inspired by this work, Gao et al. fabricated a tubular micromotor featuring a Pt-coated inner surface and an inert outer layer, similarly propelled by O_2_ bubbles from H_2_O_2_ decomposition [87]. Subsequently, Khezri et al. later adapted this tubular design for drug delivery applications (Figure 3a) [88], employing an n-rGO/Pt (reduced oxide nanoene/platinum) micromachine. These n-rGO/Pt motors demonstrated exceptional velocities even at low H_2_O_2_ concentrations, achieving an average speed of 246 µm/s in 0.5% H_2_O_2_. Furthermore, the integration of electrochemical stimulation enabled rapid, site-specific doxorubicin (DOX) release within seconds. However, the substantial size of these tubular MNMs renders them highly susceptible to clearance within the in vivo circulatory system, limiting their enrichment capacity at tumor sites. Despite this limitation, this self-propelled system marked a significant advancement toward sophisticated drug delivery platforms.

To miniaturize nanomotors for biomedical applications, Tu et al. employed a supramolecular chemistry approach to fabricate polymer-based nanomotors known as stomatocytes (Figure 3b) [89]. These bowl-shaped structures were formed through the controlled shape transformation of the block copolymer poly(ethylene glycol)-b-polystyrene (PEG-b-PS). A key feature is the opening on one side, which provides the necessary asymmetry for nanomotor propulsion. Platinum nanoparticles (Pt NPs) were encapsulated within the stomatocytes to serve as the power source. Catalytic decomposition of H_2_O_2_ by the Pt NPs generates O_2_ bubbles, propelling the motors forward at speeds of up to 35 μm/s in the presence of 4.98 mM H_2_O_2_. Furthermore, the polymeric vesicles incorporate disulfide bonds, conferring reduction-responsive properties. In the presence of glutathione (GSH), these bonds are reduced, causing the vesicles to disassemble and release any encapsulated drug. This work successfully integrates nanomotor propulsion with a reduction-responsive drug release mechanism, overcoming limitations of traditional drug delivery control. The disulfide bonds not only ensure structural stability but also enhance biodegradability, offering novel insights for developing intelligent drug delivery systems. Looking ahead, this design principle can be extended to other stimuli-responsive systems, such as pH-sensitive or enzyme-responsive variants. It holds significant promise for applications like tumor-targeted therapy and precision drug delivery at inflammatory sites. Due to the biotoxicity of metal-based catalysts, enhancing biocompatibility remains a key challenge. To address this, natural catalase (CAT) has also been integrated into MNMs. For example, Gao et al. developed pH-sensitive biocatalytic porous micromotors by co-embedding β-lactoglobulin and catalase within zeolitic imidazolate framework-L (ZIF-L) (Figure 2c) [90]. This design enabled reversible movement control at ultra-low physiological H_2_O_2_ concentrations (98–980 μM) through pH-triggered allosteric transformation of β-lactoglobulin.

Recently, Lv et al. designed a nanomotor consisting of a titanium dioxide (TiO_2_) core enclosed within periodic mesoporous organosilica (PMO) rods modified with CAT, termed HTiPC (Figure 3d) [91]. These self-propelled nanomotors utilize excess H_2_O_2_ as fuel to enhance tissue penetration and improve PDT efficacy. The motion mechanism arises from asymmetric ionic concentration gradients generated by enzymatic reactions on the CAT-modified PMO subunit surface. This drives movement towards the HTiO_2−_x component via ionic self-diffusiophoresis. In 200 μM H_2_O_2_, the HTiPC nanomotors achieved an average velocity of 18.71 μm/s and a diffusion coefficient of 4.6 μm^2^/s. Furthermore, by decomposing endogenous H_2_O_2_ within solid tumors, HTiPC continuously supplies O_2_. This oxygen generation facilitates efficient ROS production by black-TiO_2_ under NIR photocatalysis, thereby amplifying PDT. As shown in Figure 3e, Yan et al. developed a controllable super-assembly approach to synthesize tadpole-shaped MNMs, designated AHOASTs [92]. These MNMs comprise a catalase-functionalized Au nanosphere head and a silica hollow tail. In a solution containing 1.5% H_2_O_2_, the diffusion coefficient of AHOASTs significantly increased to 3.1 μm^2^/s, compared to a Brownian-motion-limited coefficient of 1.1 μm^2^/s in the absence of fuel. The high structural asymmetry of these tadpole-shaped MNMs enables greater efficiency relative to spherical designs [38,93]. Regarding self-electrophoresis-driven MNMs, Li et al. introduced an H_2_O_2_-driven Janus Au nanorod-Pt nanomotor (JAuNR-Pt) (Figure 3f) [41]. This nanomotor is intended for enhanced deep-tumor NIR-II photoacoustic imaging (PAI) and Pt^2+^-mediated chemotherapy. Self-propulsion in the presence of H_2_O_2_ promotes cellular uptake, accelerates lysosomal escape, and facilitates sustained Pt^2+^ ion release within the cell nucleus, inducing DNA damage and apoptosis.

#### 2.1.2. Glucose-Dependent MNMs

Glucose serves as a vital nutrient and the primary energy source for human cells. Its concentration in the bloodstream typically ranges from 3.9 to 7.1 mM, fluctuating dynamically throughout the day [94]. Glucose is also crucial for the design of MNMs, as H_2_O_2_—a key metabolic product of glucose—powers their propulsion. Enzymes catalyzing this reaction are typically glucose oxidase (GOx) or glucose oxidase-like nanozymes (e.g., AuNPs). These enzymes are often integrated with catalase (CAT) or inorganic catalysts to enable cascade catalytic reactions, thereby generating propulsive force for MNMs. Consequently, developing efficient GOx-CAT dual-enzyme cascade propulsion systems represents a major research focus for blood-based delivery platforms. This approach is particularly valuable in TME, where low endogenous glucose concentrations limit H_2_O_2_ availability. Here, GOx supplementation enhances local H_2_O_2_ levels, ensuring reliable operation of H_2_O_2_-dependent MNMs in therapeutic applications.

**Figure 4:**
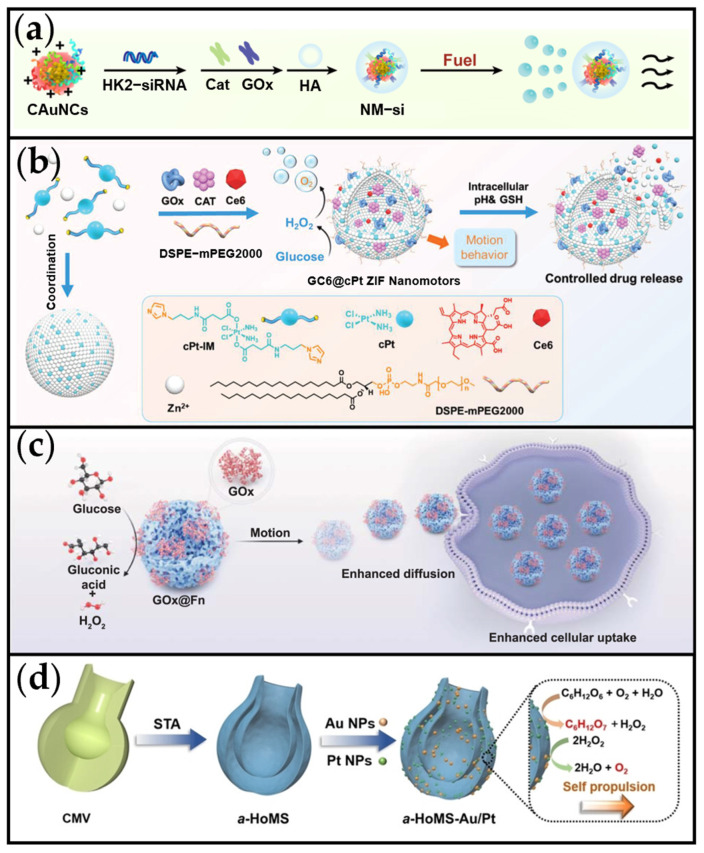
Glucose-dependent MNMs. (**a**) Schematic of the cascade enzyme-powered nanomotor (NM-si) for TME modulation (reproduced from [96] with permission from Elsevier). (**b**) Cisplatin-backboned ZIF nanomotors for encapsulation and dual-responsive release (reproduced from [97] with permission from John Wiley and Sons). (**c**) Schematic illustrating the motion behavior of GOx@Fn proteomotors (reproduced from [98] with permission from IOPSCIENCE). (**d**) Fabrication process and catalytic mechanism of a-HoMS-Au/Pt nanomotors (reproduced from [99] with permission from John Wiley and Sons).

Mano et al. pioneered the first demonstration of an artificial system driven by GOx in conjunction with bilirubin oxidase (BOD) [95]. Subsequently, MNMs utilizing GOx/CAT emerged, including motors with potential for cancer treatment. Yu et al. developed a glucose-dependent nanomotor (NM-si) designed to enhance drug penetration and therapeutic efficacy within the TME (Figure 4a) [96]. This self-propelled NM-si was constructed by co-adsorbing GOx and CAT onto positively charged Au nanoclusters. Within the system, GOx catalyzes the oxidation of intratumoral glucose, producing gluconic acid and H_2_O_2_. In the subsequent step, CAT decomposes the H_2_O_2_ into O_2_. This sequential enzymatic reaction drives the continuous generation of O_2_ bubbles, enabling the MNMs to achieve self-propulsion at a velocity of 25.25 ± 0.33 µm/s. In addition, as shown in Figure 4b, we developed a prodrug system based on cisplatin-skeletal zeolitic imidazolate frameworks (GC6@cPt ZIFs), propelled via bubble generation. This system achieved a drug loading efficiency of 13.52% [97]. The nanomotors integrated GOx, CAT, and the photosensitizer chlorin e6 (Ce6) within the ZIF, enabling self-propulsion through an enzymatic cascade that converts glucose and endogenous H_2_O_2_ into O_2_ bubbles. With an average size of 100 nm, GC6@cPt ZIFs reached a maximum speed of 2 μm/s in 10 mM glucose solution and maintained 1.4 μm/s in 5 mM glucose solution. This rapid mobility enhanced tumor accumulation following intravenous administration. Upon reaching tumor cells, the cPt ZIF framework disintegrated in response to the acidic tumor microenvironment (pH 6.5–6.9) and reductive glutathione (GSH), releasing both chemotherapeutic cPt and co-loaded cargoes. Beyond the dual-enzyme cascade system, GOx alone can propel the motor via self-diffusiophoresis. Xu et al. developed a self-propelled nanomotor (GOx@Fn) comprising GOx and ferritin (Fn) to enhance tumor therapy through ferroptosis mechanisms (Figure 4c) [98]. In this system, GOx catalyzes glucose decomposition into gluconic acid and H_2_O_2_, driving enhanced diffusion of the nanomotor. However, this approach exhibits limited motion performance, achieving an average speed of only 4.27 µm/s even in a high-concentration glucose solution (400 mM).

In addition to native enzymes, glucose-powered MNMs can also be propelled by cascades of nanoenzymes. Gao et al. engineered carbonaceous nanoflasks (CNFs) capable of autonomous propulsion in glucose-rich environments; their directional movement can be programmed through precise modulation of the nanoflasks’ surface hydrophobicity and hydrophilicity [100]. Based on this work, Hou et al. recently developed anisotropic hollow multishell structures (a-HoMS) decorated with Au/Pt nanoparticles (a-HoMS-Au/Pt) (Figure 4d) [99]. These structures exhibit glucose-driven chemotaxis and sequential drug delivery capabilities, enhancing intratumoral therapeutic efficacy. Specifically, the chemotherapy drug loaded within the a-HoMS creates a glucose concentration gradient within the TME. Subsequently, the a-HoMS-Au/Pt demonstrates directed movement towards regions of higher glucose concentration via enzyme-mediated chemoattractant behavior. Following the eradication of localized tumor cells, the a-HoMS autonomously migrates towards viable tumor cells under the persisting glucose gradient, initiating another cycle of targeted drug delivery and chemotaxis. This chemotactic behavior significantly enhances intratumoral transport and therapeutic effectiveness.

#### 2.1.3. Arginine-Dependent MNMs

Nitric oxide (NO) is a pleiotropic regulator playing critical roles in diverse biological processes, including vasodilation, neurotransmission, anti-inflammation, and immune defense [101,102]. Significantly, studies demonstrate that within the tumor microenvironment (TME), NO reacts with superoxide anions (O_2_^•−^) to form peroxynitrite (ONOO^−^), which induces the activation of matrix metalloproteinases (MMPs) in the tumor stroma [103,104]. MMPs can degrade nearly all collagen components in the extracellular matrix (ECM), thereby enhancing the penetration of therapeutic drugs into the tumor site [105,106]. Furthermore, high concentrations of NO have been shown to mediate cancer cell apoptosis and inhibit tumor growth [101]. Consequently, NO has emerged as a promising molecule for GT [107]. The primary physiological NO production pathways involve the NOS-catalyzed conversion of arginine to NO or the reaction of ROS with arginine to generate NO [78,101]. Given that NOS and ROS concentrations in the TME are substantially higher than in normal cells [78], MNMs containing arginine demonstrate enhanced propulsive mobility specifically within the TME. This nanobubble-based propulsion actuation strategy represents the predominant approach for developing NO-driven nanomotors [78,105,108,109,110].

**Figure 5:**
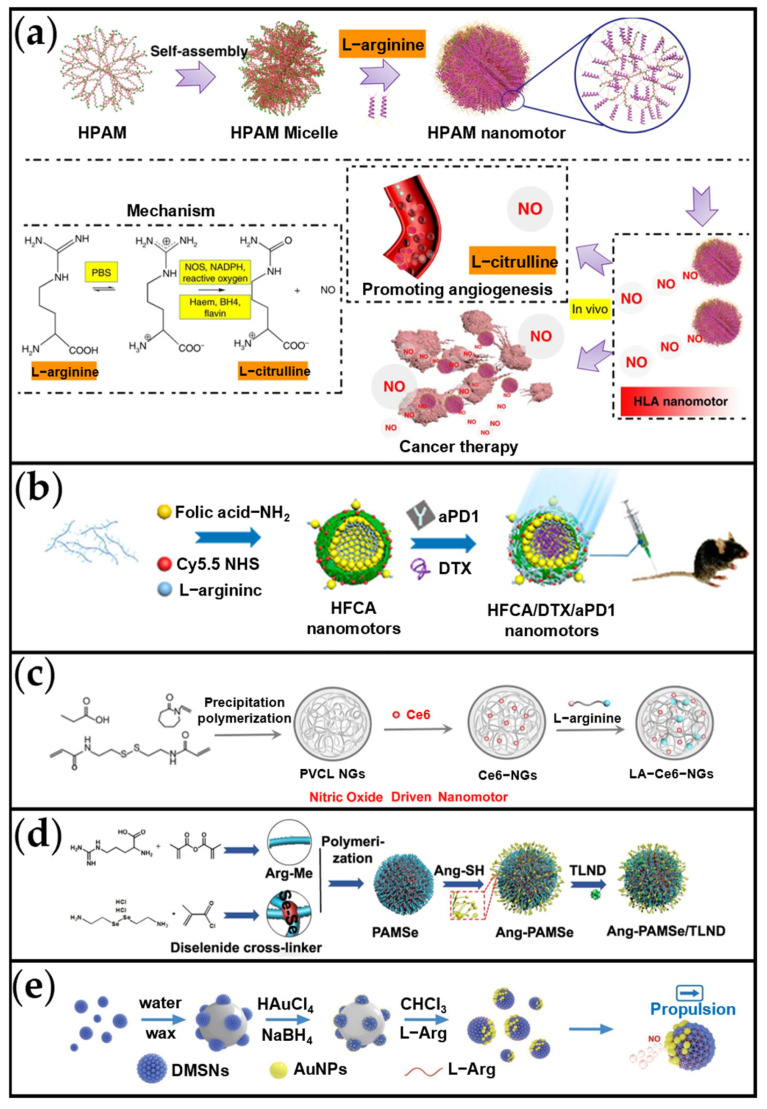
Arginine-dependent MNMs. (**a**) Schematic illustration of the formation of zwitterion-based nanomotor and the NO generation principle (reproduced from [78] with permission from Springer Nature). (**b**) Synthetic route of HFCA/DTX/aPD1 nanomotor (reproduced from [110] with permission from Journal of the American Chemical Society). (**c**) Synthetic route of LA-Ce6-NGs nanomotor (reproduced from [109] with permission from Elsevier). (**d**) Preparation process of PAMSe/TLND nanomotor (reproduced from [108] with permission from Springer Nature). (**e**) Schematic diagram of the preparation of AG-DMSNs and schematic diagram of the motion of AG-DMSNs driven by produced NO (reproduced from [112] with permission from John Wiley and Sons).

Wan et al. developed an arginine-based nanomotor (HLA nanomotor; Figure 5a [78]) representative of NO-driven propulsion systems. Synthesized via electrostatic self-assembly of hyperbranched polyamidoamine (HPAM) with arginine, this nanomotor generates beneficial byproducts (e.g., the antioxidant L-citrulline) without yielding toxic residues. The positively charged arginine readily aggregates with negatively charged molecules, facilitating nanoparticle formation. The size of these MNMs was tunable from 120 to 385 nm and exhibited a proportional increase with arginine concentration. In 20% H_2_O_2_ solution, velocities ranged from 3 to 13 μm/s and correlated positively with particle size. This electrostatic assembly approach demonstrates broad applicability: materials such as chitosan, polylysine, and heparin/folic acid can similarly self-assemble with arginine to form NO-driven nanomotors. Such versatility enables functionalization for diverse biological applications. For instance, folic acid-modified nanomotors enhance cancer cell targeting specificity, thereby improving therapeutic efficacy [111]. In a related study, Chen et al. (Figure 5b [110]) engineered an NO-driven heparin/folic acid-arginine nanomotor (HFCA) for cancer treatment. The HFCA platform was loaded with the chemotherapeutic docetaxel (DTX) and the immune checkpoint inhibitor anti-PD1. This system enhances targeted drug delivery and deep penetration into tumor tissues, achieving synergistic chemo-immunotherapy.

Beyond its capacity for self-assembly with other positively charged molecules, positively charged arginine can also be readily adsorbed by certain negatively charged delivery carriers. As depicted in Figure 5c, Wang et al. developed an NO-driven nanomotor (LA-Ce6-NGs) to enhance tumor penetration and SDT efficacy [109]. Synthesized via precipitation polymerization, this poly(N-vinylcaprolactam)-based nanogel co-encapsulates the sonosensitizer chlorin e6 (Ce6) and arginine through hydrophobic and electrostatic interactions. In solutions containing H_2_O_2_ (1–5 wt%), LA-Ce6-NGs exhibited directional movement, with velocity increasing from 4 to 11 μm/s as H_2_O_2_ concentration rose. Polymerization provides a rapid, straightforward method for nanoparticle synthesis and can be directly employed for fabricating NO-driven nanomotors. Similarly, Chen et al. fabricated arginine-based nanoparticles (PAMSe) via free radical polymerization and modified their surface with a targeting peptide, creating NO-driven nanomotors (PAMSe/TLND) to improve glioblastoma immunotherapy efficacy (Figure 5d) [108]. These nanomotors exploited elevated ROS and NOS levels in the TME to generate NO, enabling self-propulsion at 5.2 ± 1.0 μm/s. Upon crossing the blood–brain barrier and entering cells, released TLND disrupted mitochondrial metabolism, while generated NO induced immunogenic cell death. This dual mechanism significantly enhanced immunotherapy outcomes.

The NO-driven nanomotors previously described typically rely on NOS or reactive ROS to initiate their operation. Nevertheless, through rational design, NO-driven nanomotors can achieve self-propulsion even in environments with insufficient endogenous NOS or ROS levels. A prime example involves leveraging enzymatic cascade reactions: GOx catalyzes the generation of H_2_O_2_, which further oxidizes arginine to produce NO, thereby providing the thrust required for nanomotor propulsion. Building on this concept, Zheng et al. developed a self-propelled nanomotor system (AG-DMSNs) that utilizes cascade catalytic reactions to autonomously release NO, enabling enhanced penetration and eradication efficacy against methicillin-resistant *Staphylococcus aureus* (MRSA) biofilms (Figure 5e) [112]. In this design, AuNPs mimic the activity of GOx to generate H_2_O_2_, which then drives the oxidation of arginine to produce NO through a sequential catalytic process. Propelled by NO bubbles, this asymmetric nanomotor structure exhibited a remarkable movement speed of 10.9 μm/s in a 1% glucose solution. Beyond biofilm remediation, the self-propulsion mechanism of AG-DMSNs holds significant promise for cancer therapy. This system effectively harnesses the elevated expression of NOS and ROS within the TME while additionally deriving propulsion from glucose, a nutrient abundant in biological fluids. Notably, the prevalence of glucose in blood further expands its potential applications, including targeted drug delivery in circulatory systems. Collectively, these enzyme-cascade-driven nanomotors not only address the limitations of traditional NO-driven systems but also provide novel conceptual insights for the development of next-generation smart nanomachines with broad biomedical utility.

#### 2.1.4. Urea-Dependent MNMs

Urea is the final product of protein metabolism in the human body [113]. After being synthesized in the liver, urea enters the bloodstream directly and is subsequently transported to the kidneys for excretion via urine [114]. The urea concentration in normal human blood typically ranges from 2.86 to 7.14 mM, while in urine it varies, ranging from 275 to 409 mM [113,115]. As a substrate for urease, urea undergoes catalytic decomposition into CO_2_ and NH_3_. Both products are soluble in water, forming ionic salts, enabling urea to function as a fuel for urease-powered MNMs. Numerous urease-powered MNMs have been developed to date, including Janus and pot-like structures [38,116,117,118]. These MNMs achieve effective locomotion via an electrolyte self-diffusiophoresis mechanism in aqueous solutions containing physiological concentrations of urea [117]. For cancer treatment applications, urea-dependent MNMs exhibit high motility, making them highly promising candidates for injectable motors within minimally invasive, targeted theranostic platforms operating in the bloodstream. Furthermore, urea-dependent MNMs offer unique advantages for the specific treatment of bladder cancer [119].

**Figure 6:**
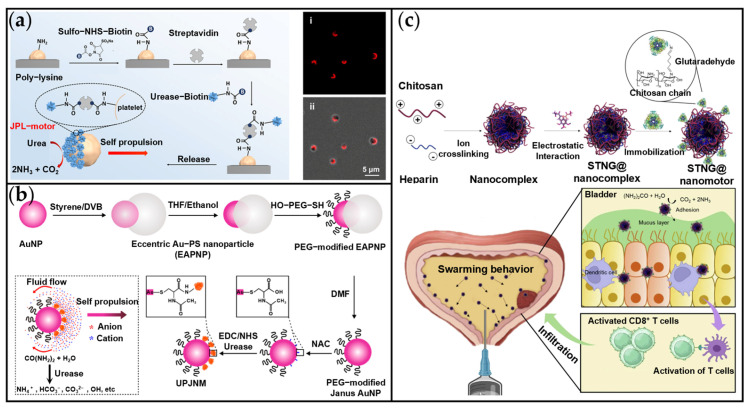
Urea-dependent MNMs. (**a**) Schematic illustrating the fabrication of JPL motors and Cy5-labeled urease-conjugated JPL motors. Fluorescent (i) and merged (ii) images of Cy5 and bright-field channels of JPL-motors with Cy5-labeled urease (reproduced from [52] with permission from The American Association for the Advancement of Science). (**b**) Schematic outlining the fabrication procedure of UPJNMs. Inset: Propulsion mechanism of the UPJNM (reproduced from [121] with permission from Journal of the American Chemical Society). (**c**) Preparation procedure for urease-powered nanomotors loaded with STING agonist via chitosan/heparin electrostatic interaction and their intravesical delivery for bladder cancer immunotherapy (reproduced from [122] with permission from Springer Nature).

Tang et al. developed a urease-driven Janus micromotor (JPL-motor) to enhance targeted drug delivery (Figure 6a) [52]. Utilizing a polylysine surface-binding strategy to asymmetrically immobilize urease on natural platelets, the JPL-motor achieved self-propulsion via urea decomposition. This decomposition generated gradients of NH_3_ and CO_2_, enabling chemotactic motion in various biofluids (PBS, serum, cell medium) at speeds 1.62 times faster than their non-Janus counterparts. In vitro experiments demonstrated that JPL-motors loaded with the anticancer drug DOX bound to MDA-MB-231 cells (Human breast cancer cells) more rapidly: within 10 min, their associated fluorescence intensity increased 2.55-fold compared to passive systems. Moreover, these active JPL-motors reduced MDA-MB-231 cell viability to 40%, a significant enhancement over the 70% viability observed with passive systems. This platform eliminates reliance on external energy sources and toxic fuels, offering a versatile and innovative strategy for active drug delivery with promising applications in cancer therapy. Since nanoparticle accumulation in tumors is optimal within the 30–200 nm size range [120], overcoming biological barriers necessitates miniaturization. To achieve autonomous motion within the bloodstream, Yang et al. developed ultrasmall enzyme-powered Janus nanomotors (UPJNMs) (Figure 6b) [121]. Using a scalable colloidal chemistry approach, they asymmetrically functionalized Janus-type Au-polystyrene nanoparticles (30–100 nm in size), coating one hemisphere with PEG brushes and immobilizing urease on the other. Urease enzymatically catalyzed the decomposition of urea into NH_4_^+^ and CO_3_^2−^ ions, establishing an ion gradient that propelled the nanomotor via self-diffusion electrophoresis. Significantly, the UPJNMs exhibited sustained mobility at physiological urea concentrations (5–10 mM) and displayed remarkable ionic tolerance in complex biological fluids (e.g., PBS, serum). These properties were attributed to their ultrasmall dimensions and protective PEG surface modification.

Given that urea concentrations in urine can reach as high as 275 to 409 mM, urea-dependent nanomotors (MNMs) have been frequently employed in bladder cancer therapeutic research [122,123]. As illustrated in Figure 6c, Choi et al. engineered urea-driven nanomotors (STING@nanomotor) composed of chitosan-heparin electrostatic complexes encapsulating a STING agonist, designed to enhance immunotherapy for bladder cancer [122]. Within the urinary environment, the STING@nanomotor utilizes urease to catalyze the hydrolysis of urea into CO_2_ and NH_3_. This reaction generates a propulsive force that facilitates penetration through the protective glycosaminoglycan layer lining the bladder. These nanomotors (600–800 nm in size) achieved a high drug loading efficiency of 83.5% and enabled sustained, pH-responsive release of the STING agonist. Compared to the control group and free STING agonist (which showed 28.5% suppression), STING@nanomotor treatment resulted in a remarkable 94.2% suppression of tumor growth. Furthermore, it significantly enhanced CD8^+^ T cell infiltration, achieving an 11.2-fold increase. By efficiently delivering the STING agonist, this urease-powered nanomotor activates the immune response and remodels the tumor microenvironment. Consequently, it significantly inhibits bladder cancer progression and provides a robust theoretical and practical foundation for clinical translation.

#### 2.1.5. Other Chemically Driven MNMs

**Figure 7:**
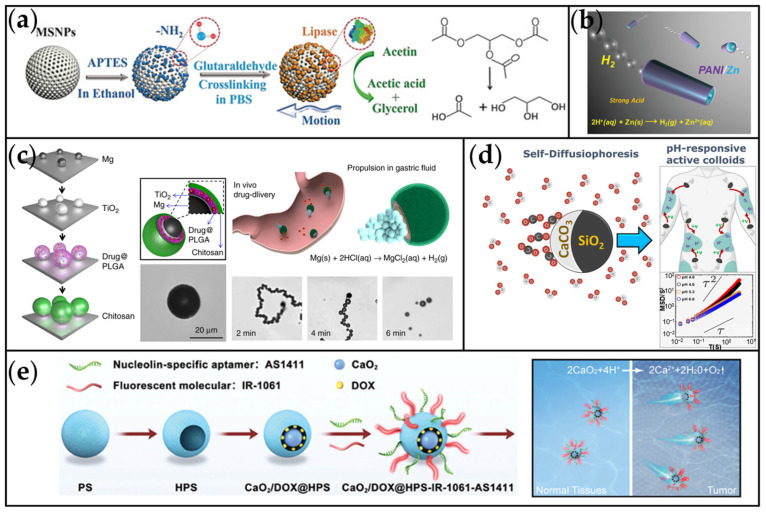
Other chemically driven MNMs. (**a**) Schematic representation of the functionalization strategy for preparing lipase-based nanomotors (reproduced from [124] with permission from John Wiley and Sons). (**b**) Schematic of motion in an acidic environment (reproduced from [126] with permission from Journal of the American Chemical Society). (**c**) Schematic preparation of Mg-based micromotors and in vivo propulsion and drug delivery in a mouse stomach (reproduced from [40] with permission from Springer Nature). (**d**) pH-responsive Janus CaCO_3_ micromotors (reproduced from [127] with permission from Journal of the American Chemical Society). (**e**) Schematic illustration of the sequential synthesis of CaO_2_/DOX and degradation of CaO_2_ (reproduced from [128] with permission from John Wiley and Sons).

As shown in Figure 7a, Wang et al. developed lipase-based nanomotors capable of sustaining enhanced Brownian motion in triglyceride solutions for extended durations [124]. These nanomotors operate via an enzymatic propulsion mechanism, utilizing product gradients generated through the degradation of triglyceride molecules. Under normal physiological conditions, where blood triglyceride concentrations reach 1.7 mM [125], these nanomotors can access adequate fuel directly in the bloodstream. Consequently, as drug delivery vehicles, they are thereby expected to enhance drug accumulation in tumor regions.

Beyond enzymatic reactions, reactive metals like Zn and Mg can also power micromotors by reducing H^+^ to H_2_ in strong acids [126,129,130]. The low solubility of H_2_ in water results in abundant bubble generation, propelling the motor forward. For example, Gao et al. developed tubular polyaniline (PANI)/Zn micromotors featuring Zn coatings on both the inner and outer tube surfaces (Figure 7b) [126]. In acidic environments, the reaction Zn + 2H^+^ → Zn^2+^ + H_2_ ↑ generates H_2_ bubbles on the Zn surface, enabling propulsion speeds exceeding 100 body-lengths per second. Due to their acid dependence, such motors are often suitable for gastric drug delivery applications [129]. Building on this concept, Ávila et al. employed Mg-based micromotors loaded with the antibiotic clarithromycin to target *Helicobacter pylori* infections in a murine model (Figure 7c) [40]. These Mg micromotors utilize gastric acid as a fuel source for self-propulsion (achieving an average speed of 120 μm/s in simulated gastric fluid) while simultaneously neutralizing local acidity via the proton-consuming reaction Mg + 2H^+^ → Mg^2+^ + H_2_ ↑. This dual action eliminates the need for proton pump inhibitors and significantly inhibited *H. pylori* proliferation in studies, suggesting a potential role in gastric cancer prevention [131]. The mobility and acid-neutralizing capabilities of Mg-based micromotors highlight their potential for broader gastric cancer therapeutics.

In acidic solutions, CaCO_3_ decomposes to generate ion gradients that can power micromotors. For example, Saad et al. fabricated pH-responsive Janus CaCO_3_ micromotors using the Pickering emulsion method (Figure 7d) [127]. At pH 4.0, controlled decomposition of CaCO_3_ into Ca^2+^ and HCO_3_^−^ enabled propulsion via self-diffusion mechanisms, achieving an average velocity of 3.2 μm/s (reaction: CaCO_3_ + 2H^+^ → Ca^2+^ + H_2_O + CO_2_). These Janus CaCO_3_ MNMs demonstrated excellent biocompatibility and can be leveraged for drug loading and pH-gradient-triggered targeted release. Similarly, CaO_2_ decomposes in acidic environments to produce substantial amounts of oxygen (reaction: 2CaO_2_ + 4H^+^ → 2Ca^2+^ + 2H_2_O + O_2_ ↑) [132]. Based on this characteristic, Lu et al. designed a self-propelled nanomotor system (CaO_2_/DOX@HPS-IR-1061-AS1411) with CaO_2_ as the driving core for precise tumor combination therapy (Figure 7e) [128]. This system autonomously propels within the acidic TME, specifically targets 4T1 cancer cells, and enhances the therapeutic efficacy of DOX. This approach could extend to other metal peroxide systems. Further exploration of its integration with complementary therapeutic modalities may establish a new paradigm for precision tumor treatment.

### 2.2. Physically Driven MNMs

#### 2.2.1. Light-Driven MNMs

Light-driven MNMs, which utilize specific wavelengths of light for motion control, are particularly well-suited for photoresponsive actuation within biological systems [67]. Near-infrared light (NIR) is especially advantageous due to its exceptional biocompatibility and deep tissue penetration capabilities [133], making it an optimal stimulus for MNMs in cancer therapy [134]. The motion mechanism of NIR-driven MNMs primarily relies on the generation of a thermal gradient through photothermal effects; this gradient subsequently propels the MNMs via self-thermophoresis [135]. The successful development of these NIR-responsive MNMs provides new opportunities to enhance treatment precision and minimize off-target effects in cancer therapy. For cancer treatment applications, NIR-driven MNMs are predominantly fabricated using semiconducting polymers (SP) [136,137,138,139] polydopamine (PDA) [140,141,142], and metal nanoparticles [143,144,145], among other materials.

**Figure 8:**
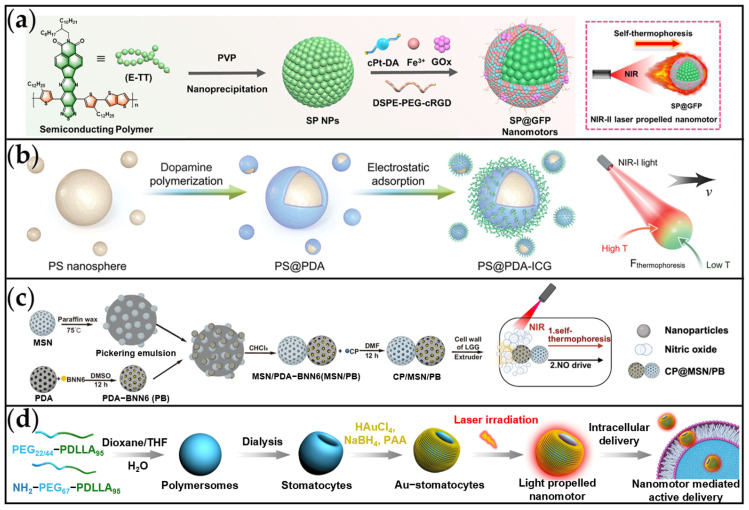
Light-driven MNMs. (**a**) Schematic of a core–shell SP@GFP nanomotor’s construction and its propulsion via near-infrared (NIR) laser-controlled self-thermophoresis(reproduced from [146] with permission from Elsevier). (**b**) Schematic illustrating the fabrication process of the PS@PDA-ICG nanomotor and its thermophoretic mechanism (reproduced from [147] with permission from John Wiley and Sons). (**c**) Schematic of the CMPBC synthetic procedure and its movement direction under NIR irradiation (reproduced from [148] with permission from Springer Nature). (**d**) Schematic illustrating the preparation of light-propelled biodegradable stomatocyte nanomotors (reproduced from [149] with permission from Springer Nature).

We have successfully engineered a near-infrared-II (NIR-II) light-driven core–shell prodrug nanomotor (SP@GFP), utilizing a self-thermophoretic mechanism to achieve nanomotor motion (Figure 8a) [146]. The SP@GFP comprises a NIR-II-absorbing (1000–1700 nm) SP core, encapsulated within a metal-phenolic network (MPN) shell co-loaded with a cisplatin prodrug (cPt-DA) and GOx. Under 1064 nm laser irradiation, the nanomotors exhibited efficient photothermal conversion (46.1% efficiency; resulting in a 25 °C temperature increase) and sustained self-thermophoretic motion (4.4 μm/s at 1.5 W/cm^2^). Critically, the photothermally driven thermophoresis of the SP core significantly enhanced deep-tissue penetration, thereby boosting tumor accumulation and penetration depth within tumor spheroids. Through the rational design of this nanomotor combined with NIR-II remote control, the SP@GFP platform successfully integrates PTT, chemotherapy, and catalytic ferroptosis induction, presenting a highly promising strategy for synergistic cancer treatment.

As a common photothermal material, PDA exhibits good biocompatibility and plasticity, making it well-suited for fabricating photothermal motors in various shapes [140,141,142]. Jiang et al. designed a NIR-II fluorescent thermophoretic nanomotor (PS@PDA-ICG) using a template-based method (Figure 8b) [147]. This nanomotor integrated a polystyrene (PS) core, a PDA shell, and surface-anchored indocyanine green (ICG), allowing synergistic NIR-I (808 nm) photothermal conversion and real-time NIR-II fluorescence imaging. Upon NIR-I irradiation, the nanomotor demonstrated photocontrolled thermophoretic motion with velocities up to 10 μm/s at an intensity of 0.5 W/cm^2^. This motion facilitated penetration through subcutaneous barriers (such as fat and collagen) via localized photothermal ablation. PS@PDA-ICG thus holds potential for efficient and low-risk peritumoral PTT for superficial tumors when administered via localized injection. Wang et al. developed a dual-actuated bioactive nanomotor (CMPBC) engineered to overcome intestinal barriers for the oral treatment of colorectal cancer (CRC) (Figure 8c) [148]. The CMPBC nanomotor features a Janus double-sphere structure: one sphere consists of mesoporous silica loaded with cisplatin, while the other is a PDA sphere serving as an NO donor via BNN6 encapsulation. The structure is further coated with a lactic acid bacteria cell wall to enable IgA-mediated CRC targeting. Under NIR laser irradiation, CMPBC generates self-thermophoresis and NO-driven propulsion, allowing rapid penetration of the intestinal mucus barrier. This significantly enhances drug delivery efficiency and facilitates effective CRC therapy.

Wang et al. developed a light-driven nanomotor platform using asymmetric, bowl-shaped polymer vesicles (stomatocytes) modified with Au NPs (Figure 8d) [149]. The photothermal effect of the Au NPs under 660 nm or 808 nm laser irradiation generated asymmetric plasmonic heating, producing a thermophoretic force that propelled the stomatocytes with an average velocity of 124.7 ± 6.6 μm/s. This enabled rapid, high-speed, and precisely controllable motion. Constructed from biocompatible PEG-PDLLA, these nanomotors leveraged their controllable photothermal properties to deliver cargoes (FITC-BSA, Cy5-siRNA, and doxorubicin) into living cells through membrane disruption. Crucially, within a 3D HeLa tumor spheroid model, laser irradiation significantly enhanced the drug penetration depth compared to non-irradiated controls. This study establishes a robust and versatile platform for light-driven, biodegradable nanomotors, demonstrating their significant potential for targeted drug delivery and diverse biomedical applications.

#### 2.2.2. Magnetic-Driven MNMs

Magnetic driving technology enables precise control and navigation of MNMs using external magnetic fields [150]. Studies have demonstrated the transportation capabilities and characteristics of magnetically propelled MNMs [151,152,153,154]. Furthermore, research on magnetic-driven MNMs for cancer treatment has yielded promising results. A fundamental requirement for this propulsion mechanism is that MNMs must be both magnetically responsive and helically shaped. These helices convert rotational torque generated by an external magnetic field into translational motion [155,156,157]. Magnetic actuation also offers advantages including rapid responsiveness, remote non-contact control, safe human–machine interaction, and cost-effectiveness [158].

**Figure 9:**
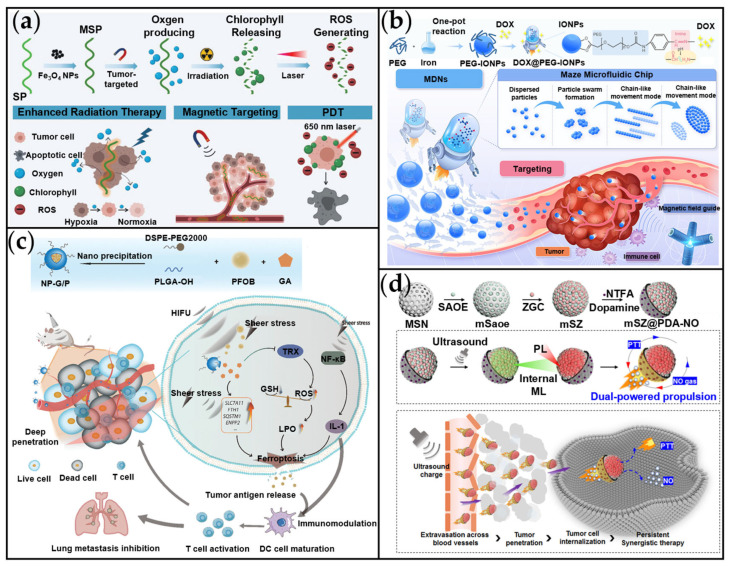
Magnetic-driven MNMs and ultrasound-driven MNMs. (**a**) Schematic illustration of magnetic *S. platensis*-based microswimmers for tumor targeting, enhanced radiation therapy, and combined photodynamic therapy (reproduced from [159] with permission from John Wiley and Sons). (**b**) Synthesis of MDNs and their active drug delivery in vivo via physical targeting (reproduced with [34] from permission from Elsevier). (**c**) Synthetic procedure of NP-G/P and the antitumor mechanism of HIFU-driven nanomotors against TNBC, leveraging improved tumor penetration and robust ferroptosis-immunotherapy (reproduced from [160] with permission from John Wiley and Sons). (**d**) Synthesis of Janus mSZ@PDA-NO NPs. Under internal NIR excitation, the PDA caps generate a persistent self-thermophoretic force to propel MNMs motion, enhancing tumor accumulation, intratumoral penetration, and intracellular uptake (reproduced from [142] with permission from the American Chemical Society).

An important synthesis method for magnetic-driven MNMs is the bio-template approach. This method integrates suitable microorganisms with magnetic nanomaterials to fabricate bio-hybrid micro/nanorobots. Zhong et al. developed a photosynthetic biohybrid nanoscale swimmer system (PBNs) using magnetically engineered *Spirulina platensis* (*S. platensis*). Designed to alleviate tumor hypoxia and enable multimodal imaging-guided synergistic therapy (Figure 9a) [159]. Magnetic *S. platensis* (MSP) was developed by attaching superparamagnetic Fe_3_O_4_ nanoparticles to native *S. platensis*. The fabricated MSP could be subjected to controlled actuation due to the magnetic properties of Fe_3_O_4_ nanoparticles. The MSP facilitated precise magnetic-driven navigation (achieving speeds of up to 78.3 μm/s) and significantly enhanced tumor accumulation under magnetic guidance in vitro. In vivo studies demonstrated robust tumor suppression through the synergy of radiotherapy (RT) and PDT, with minimal systemic toxicity. The integration of biocompatible spirulina, dynamic oxygen modulation, and theranostic multifunctionality offers a transformative platform for hypoxia-resistant, imaging-guided cancer therapy.

Furthermore, individual magnetic nanoparticles can also serve as drug-carrying nanomotors. Wang et al. developed magnetically driven bioinspired nanorobots (MDNs) loaded with DOX for enhanced tumor therapy, leveraging both targeted drug delivery and immune activation (Figure 9b) [34]. The MDNs, composed of ultra-small iron oxide nanoparticles (IONPs, 7.02 ± 0.18 nm) functionalized with PEG and pH-sensitive DOX loading (12.4% *w*/*w*), exhibited superparamagnetism (56.66 emu/g). A 3D magnetically controlled platform (MMP) facilitated the precise navigation of MDNs at a speed of 100 μm/s, replicating fish-swarm-like motion (including dispersed particles, particle swarm formation, chain-like movement, and dish-like movement) to effectively penetrate biological barriers in complex physiological environments. By integrating magnetically guided chemo-immunotherapy with bioimaging capabilities, this work establishes a pioneering strategy for precision tumor treatment, particularly beneficial for vascularized tumors, while also addressing the therapeutic challenges associated with irregular tumor morphologies.

#### 2.2.3. Ultrasound-Driven MNMs

Ultrasound provides an external energy source that drives the movement of MNMs [161]. Within static ultrasound fields, MNMs demonstrate various behaviors—including suspension, self-propulsion, rotation, and accumulation—controlled by acoustic radiation forces [68]. Establishing an acoustic condition is relatively straightforward. Since sound waves can propagate through various media, including solids, liquids, and gases, they are capable of penetrating biological tissues to provide external power to nanomotor without causing significant harm to the human body [36].

Yu et al. developed a HIFU-driven nanomotor (NP-G/P) for the effective treatment of triple-negative breast cancer (TNBC) by inducing ferroptosis (Figure 9c) [160]. The NP-G/P consisted of a PLGA matrix encapsulating gambogic acid (GA) and perfluorobromane (PFOB), with its surface further modified with DSPE-PEG. Under HIFU irradiation at 8.5 W, the PFOB exhibited pronounced cavitation effects. This effect simultaneously enhanced drug release and propelled the nanoparticle. Specifically, HIFU exposure (8.5 W, 5 min) triggered a 65.33% release of GA from NP-G/P. GA induced ferroptosis primarily by inhibiting thioredoxin (TRX), leading to glutathione depletion, elevated ROS levels, and lipid peroxidation. HIFU irradiation further amplified ferroptosis-driven immunogenicity, promoting the release of damage-associated molecular patterns (DAMPs). These DAMPs subsequently activated dendritic cells and stimulated T-cell infiltration. Together, GA and HIFU synergistically suppressed primary tumor growth and lung metastases. Ultrasound not only directly propels the nanomotor but also supplies energy for its thermophoretic motion through an energy conversion process. Zhang et al. developed an ultrasound-activated nanoplatform (mSZ@PDA-NO) for synergistic tumor therapy (Figure 9d) [142]. The mSZ@PDA-NO nanoplatform employs a core–shell structure where mesoporous silica nanoparticles (MSNs) are coated with mechanoluminescent SrAl_2_O_4_:Eu^2+^ (SAOE) and NIR-emitting ZnGa_2_O_4_:Cr^3+^ (ZGC). This design facilitates ultrasound-triggered persistent luminescence. Within the internal structure, ZGC and SAOE enable energy transfer that converts ultrasonic waves into NIR light. Simultaneously, PDA absorbs the emitted NIR light to generate a thermophoretic effect, propelling the motor at speeds of 5.5–7.4 μm/s. Nitrothiomorpholine dioxide (NTFA), acting as an NO donor, releases NO under photothermal stimulation, further enhancing propulsion. This study introduces an ultrasound-activated persistent luminescent nanoparticle enabling synergistic tumor therapy via integrated mechanisms: self-propelled motion, sustained photothermal effects, and controlled NO release.

### 2.3. Biohybrid MNMs

Biohybrid MNMs are primarily engineered by integrating live microorganisms with artificial materials [36,155]. These microorganism-powered MNMs harness the natural capabilities of microbes. For example, flagellated bacteria provide self-propulsion [162]. Additionally, anaerobic bacteria exhibit inherent tropism towards hypoxic tumor regions, making them valuable for targeted drug delivery [163]. As potent immune stimulators, bacteria can also activate the immune system, positioning bacteria-mediated tumor immunotherapy as a significant research focus [59]. Beyond bacteria, some algae offer advantages like phototaxis for targeted motion in tumor therapy, while the oxygen they generate via photosynthesis can alleviate tumor hypoxia [164].

**Figure 10:**
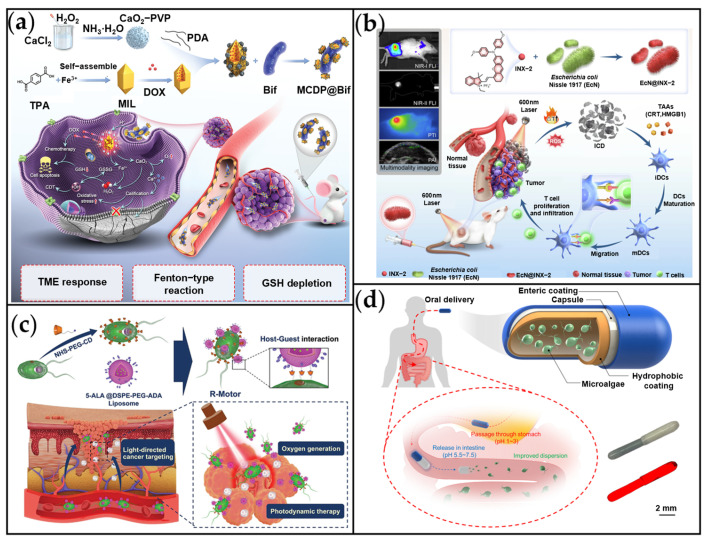
Biohybrid MNMs. (**a**) Schematic illustration of the fabrication process and anti-tumor mechanism of the biomotor MCDP@Bif (reproduced from [165] with permission from John Wiley and Sons). (**b**) Schematic illustration depicting the AIEgen bacteria hybrid bionic robot EcN@INX-2 for guiding multimodal imaging and its application in elucidating cancer immunotherapy mechanisms (reproduced from [166] with permission from Springer Nature). (**c**) Schematic illustration of the R-motor with backpacks, developed by conjugating ADA-modified liposomes (loaded with a photosensitizer) onto the surface of CD-modified *C. reinhardtii* through host-guest interactions (reproduced with [167] from permission from John Wiley and Sons). (**d**) Schematic illustration of algae motors in a capsule for gastrointestinal (GI) tract delivery (reproduced from [168] with permission from the American Association for the Advancement of Science).

Li et al. developed a biohybrid drug delivery system (MCDP@Bif) by combining anaerobic *Bifidobacterium infantis* (Bif) with MOFs containing CaO_2_ and DOX for targeted breast cancer treatment via synergistic chemotherapy and CDT (Figure 10a) [165]. Exploiting the inherent hypoxia-tropism of Bifidobacterium, the MCDP@Bif system achieved targeted accumulation within the hypoxic TME, resulting in a 3.8-fold increase in intratumoral DOX accumulation compared to free DOX. Within the TME, released Fe^2+^ ions catalyze a Fenton-like reaction utilizing H_2_O_2_ generated from CaO_2_ decomposition to produce cytotoxic •OH for CDT. Concurrently, excessive Ca^2+^ release disrupts mitochondrial function, exacerbates oxidative stress, and promotes apoptosis. This bacteria-mediated platform significantly enhanced drug enrichment within the hypoxic TME and boosted therapeutic efficacy through ROS-mediated mechanisms. Separately, Hu et al. engineered a biomimetic bacterial robot, EcN@INX-2, by integrating polymerization-induced emission luminogens (AIEgens) onto *Escherichia coli* Nissle 1917 (EcN) for synergistic phototherapy and immunotherapy of colon cancer (Figure 10b) [166]. The AIEgen, INX-2, exhibited remarkable properties including NIR-II fluorescence (emission peak at 950 nm), 46.26% photothermal conversion efficiency under 660 nm laser irradiation, and ROS generation via electron transfer. Both in vitro and in vivo experiments confirmed its potent synergistic photodynamic/photothermal effects, which successfully induced immunogenic cell death (ICD).

*Chlamydomonas reinhardtii* (*C. reinhardtii*) possesses a unique eyespot structure that enables it to sense light sources. This microalga exhibits enhanced and rapid phototaxis in environments with elevated ROS, positioning it as a promising candidate for light-driven microrobot development [169]. Exploiting this phototaxis, Wang et al. engineered a light-driven biohybrid motor system (R-motor) that significantly improves tumor treatment efficacy by integrating three key functions: targeted drug delivery, photosynthetic oxygen supply, and PDT (Figure 10c) [167]. To achieve this, *C. reinhardtii* was modified with β-cyclodextrin, enabling host-guest complexation with adamantane-functionalized liposomes loaded with the photosensitizer 5-aminolevulinic acid (5-ALA). Using red light (680 nm) to guide the algae via their natural phototaxis, the system targets tumor regions. Concurrently, algal photosynthesis generates O_2_ within the tumor microenvironment, enhancing PDT efficacy. Both in vitro and in vivo experiments demonstrated potent tumor suppression, establishing an innovative framework for microrobot applications in biomedicine. Beyond intravenous delivery, oral administration represents another common route for biohybrid microscale motors, valued for its patient compliance, non-invasiveness, and cost-effectiveness [33,170]. However, the complex gastrointestinal (GI) environment poses challenges including gastric acid degradation, inadequate intestinal wall contact, and limited drug solubility [171]. To address these limitations, Zhang et al. developed an efficient GI drug delivery platform by encapsulating the microalgae *C. reinhardtii* within a pH-responsive degradable capsule (Figure 10d) [168]. This dual-layer capsule features an inner hydrophobic coating that shields the algae from gastric acid, and an outer enteric polymer layer (Eudragit L100-55) designed to dissolve in the small intestine for targeted payload release. The algae-based motors achieved sustained speeds of ~40 μm/s at 37 °C for 12 h in intestinal fluid. In vivo studies demonstrated that motile capsules significantly enhanced intestinal payload distribution and retention compared to static algae, with a 3.5-fold increase in fluorescence intensity observed in the small intestine. Their extended motility substantially improves drug dispersion and residence within the intestinal tract, offering a promising strategy for localized GI therapies, such as inflammatory bowel disease and colon cancer. Future research could further optimize targeting strategies and evaluate therapeutic efficacy in preclinical disease models.

## 3. Targeting and Tumor Penetration of MNMs in Cancer Treatment

After entering the body, nanodrugs must sequentially accomplish five critical steps to effectively eliminate tumors: (1) maintaining stability during blood circulation, (2) achieving targeted delivery, (3) facilitating tumor accumulation, (4) enabling deep penetration, and (5) promoting cellular internalization. This intricate physiological process significantly limits the efficiency of nanomedicine delivery to the tumor site. Studies have demonstrated that merely 0.7% of nanomedicines successfully reach solid tumors following systemic administration [172,173]. Additionally, the endothelial cell barrier of tumor vasculature and the basement membrane structure of the outer vasculature (such as the blood–brain barrier) hinder the deep penetration of nanomedicines into tumors as well as their uptake by tumor cells [174,175]. Thus, overcoming the physiological barriers of tumors and enhancing targeting efficiency are two pivotal factors for improving the efficacy of tumor therapy. MNMs possess the advantages of efficient penetration and intelligent targeting capabilities. Specifically, they not only overcome physiological barriers by enhancing mechanical action in response to external fields but also function as intelligent drug carriers for targeted delivery and controlled release, thereby improving therapeutic efficacy and minimizing side effects. These unique properties make MNMs a highly promising candidate for application in tumor treatment. In this section, we provide a detailed summary of the major advancements of MNMs in intelligent targeting capabilities and in overcoming tumor physiological barriers.

### 3.1. The Targeting of MNMs

#### 3.1.1. Responsive Targeting of the Tumor Microenvironment

**Figure 11:**
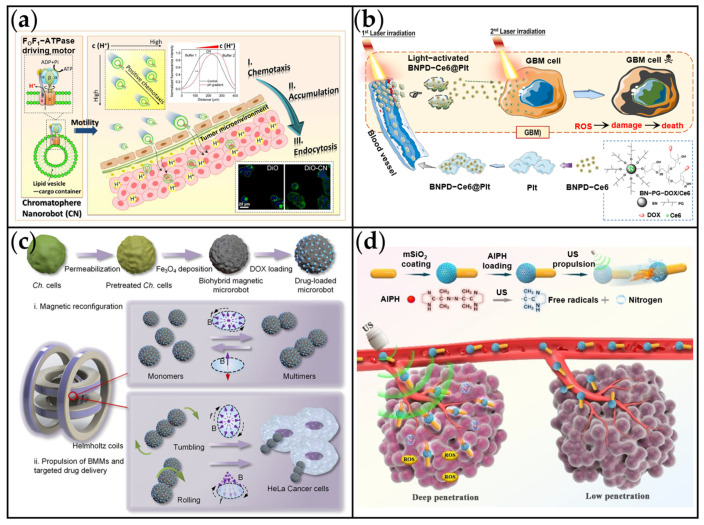
Targeted delivery strategies for MNMs. (**a**) Schematic of an F_O_F_1_-ATPase motor-embedded carbon nanomaterial (CN) designed to overcome biological barriers, exhibiting enhanced chemotaxis, strong tumor accumulation, and promoted cellular uptake (reproduced from [177] with permission from Elsevier). (**b**) Targeted photodynamic therapy of glioblastoma mediated by BNPD-Ce6@Plt (reproduced from [180] with permission from Elsevier). (**c**) Facile fabrication process of magnetic biohybrid micromotors, showcasing their magnetic reconfiguration and propulsion capabilities (reproduced from [157] with permission from the American Chemical Society). (**d**) Ultrasound-propelled Janus Au NR-mSiO_2_ nanomotor utilized for NIR-II photoacoustic imaging-guided sonodynamic-gas therapy of large tumors (reproduced from [181] with permission from Springer Nature).

Although the complexity of the TME presents significant challenges for cancer treatment, it also offers unique opportunities for targeted drug delivery [176]. Developing novel TME-targeted therapeutic strategies based on its key characteristics is crucial for optimizing anti-tumor drug design. To address this, Yang et al. engineered chromatophore-based nanomotors (CNs) that exploit the acidic TME, utilizing FoF1-ATPase biomolecular motors as the propulsion mechanism (Figure 11a) [177]. FoF1-ATPase functions as a rotary molecular motor that synthesizes ATP by harnessing the transmembrane proton gradient. This enzyme exhibits both ultra-high energy conversion efficiency (>90%) and excellent biocompatibility, efficiently transforming chemical energy into mechanical motion. Critically, its proton-driven chemotaxis enables precise targeting of acidic tumor microenvironments, underscoring its biomedical relevance [178,179]. The CNs demonstrated gradient-driven motility within the TME, migrating along proton concentration gradients to achieve significant accumulation at tumor sites. By integrating FoF1-ATPase’s self-propulsion, proton-responsive targeting, and TME specificity, this platform represents a highly promising approach for precision nanomedicine, effectively overcoming challenges in tumor-targeted drug delivery.

#### 3.1.2. Physical Field Targeting

In cancer treatment, the complex TME often suffers from insufficient intrinsic targeting drive, leading to off-target effects of MNMs [80]. To enhance tumor targeting accuracy, researchers have widely employed external energy fields—such as light, magnetic fields, and ultrasound—capable of providing stable energy outputs. For example, Xu et al. developed a light-responsive, rapid tumor-targeting drug delivery platform (BNPD-Ce6@Plt) based on platelets for the effective treatment of glioblastoma (Figure 11b) [180]. In this system, chlorin e6 (Ce6) was loaded onto boron nitride nanoparticles (BNPD) to form BNPD-Ce6. This complex was then encapsulated with platelets, yielding the final construct, BNPD-Ce6@Plt. Upon irradiation of the tumor region with an 808 nm laser, Ce6 within the platelets generated ROS, prompting rapid platelet activation and aggregation. Furthermore, BNPD’s photothermal effect facilitated the targeted release of BNPD-Ce6 from platelets into the tumor, enabling photocontrolled targeting. Following targeted BNPD-Ce6 delivery, subsequent light exposure re-triggered ROS generation, thereby producing a marked photodynamic therapeutic effect against glioblastoma. This strategy offers an approach for achieving precise, minimally invasive glioblastoma therapy via light-controlled platelet release of photosensitizers.

Gong et al. developed magnetic biohybrid MNMs based on *Chlorella* cells for enhanced targeted drug delivery (Figure 11c) [157]. These BMMs utilized approximately spherical *Chlorella* cells, typically 3–5 μm in diameter, which were magnetized through surface adsorption of Fe_3_O_4_ nanoparticles. This modification imparted superparamagnetism to the cells, exhibiting a saturation magnetization of 20.4 emu/g. The microrobots exhibited dual propulsion modes under rotating magnetic fields: rolling at 107.6 μm/s and tumbling at 72.7 μm/s under vertical field rotation. Furthermore, their pH-responsive release mechanism for doxorubicin (DOX) enabled accelerated drug release under acidic conditions, facilitating targeted chemotherapy. In vitro studies demonstrated the effective induction of apoptosis in HeLa cells through controlled targeted drug delivery enabled by a magnetic coil system. Due to their superior maneuverability in magnetic fields, these MNMs represent a highly promising platform for precise anticancer therapy.

Ultrasound (US) offers significant advantages for biomedical applications, including its non-invasiveness, low cost, ease of operation, and strong deep-tissue penetration capabilities [182,183]. These properties enable US to enhance nanoparticle-mediated disruption of physiological barriers through mechanical effects, thereby facilitating drug delivery in cancer therapy [184]. Illustrating this, Ye et al. developed a US-propelled Janus Au nanorod-mesoporous silica (Au NR-mSiO_2_/AIPH) nanomotor for deep-tissue tumor theranostics (Figure 11d) [181]. Under US irradiation, the N_2_ donor (AIPH) decomposes to generate both N_2_ bubbles and free radicals. In vivo, the nanomotors accumulated in tumors under US guidance, achieving a tissue penetration depth of 1.32 mm and significantly enhancing PA/US imaging signals. This work establishes a US-activated strategy for precise tumor targeting and noninvasive treatment of deep-seated malignancies.

#### 3.1.3. Biomimetic Targeting Strategy

Active targeting enhances nanomedicines by modifying them with specific ligands, enabling selective recognition and binding to target cells. This facilitates their active accumulation at specific sites [185]. This section describes two primary strategies for actively targeting MNMs: (1) Biological Targeting Molecule Modification: Tumor cells often overexpress specific surface receptors, providing a molecular distinction from normal cells. Modifying nanoparticle surfaces with biological ligands (e.g., antibodies, peptides, small molecules) that bind these receptors confers selective targeting towards cancer cells. Incorporating such targeting molecules significantly improves the tumor accumulation rate of nanomedicines [186]. (2) Biomembrane Camouflage: This approach involves coating nanoparticles with natural cell membranes derived from sources like cancer cells or immune cells. The resulting biomimetic nanoparticles inherit the intrinsic active targeting and homotypic (or homologous) binding capabilities inherent to these membranes [187]. Consequently, camouflaged MNMs exhibit enhanced specific targeting. Furthermore, these biomimetic strategies are often combined with TME-responsive targeting and external energy field (e.g., magnetic, ultrasound) guidance. Integrating multiple targeting modalities substantially improves both the tumor-targeting efficiency and penetration depth of MNMs.

**Figure 12:**
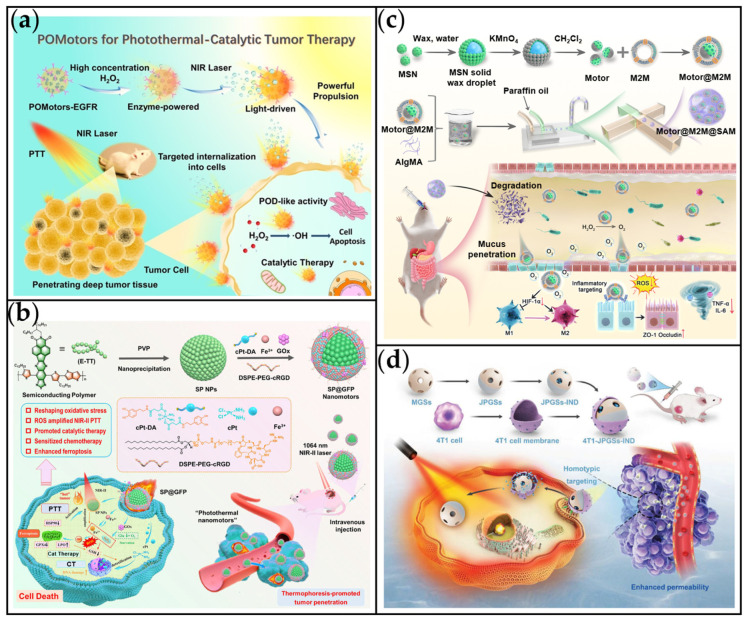
Biomimetic targeting strategy. (**a**) The scheme illustrating of POMotors for self-propulsion-promoted synergistic photothermal-catalytic tumor therapy (reproduced from [188] with permission from John Wiley and Sons). (**b**) Tailored core–shell nanomotors based on semiconducting polymer@cisplatin-skeletal metal-phenolic networks (SP@GFP) for synergistic therapy of breast cancers (reproduced from [146] with permission from Elsevier). (**c**) Schematic illustration of Motor@M2M@SAM preparation and its mechanism for urothelial carcinoma (UC) treatment (reproduced from [190] with permission from the American Association for the Advancement of Science). (**d**) Fabrication of biomimetic 4T1-JPGSs-IND and its role in enhancing PTT while alleviating PTT-induced inflammation (reproduced from [191] with permission from John Wiley and Sons).

Tang et al. engineered light-driven nanomotors (POMotors) modified with epidermal growth factor receptor antibody (anti-EGFR) for targeted synergistic photothermal-catalytic tumor therapy (Figure 12a) [188]. The epidermal growth factor receptor (EGFR) functions as a receptor for extracellular protein ligands within the epidermal growth factor family. Crucially, EGFR is overexpressed in various cancer types, including breast cancer, colorectal cancer, pancreatic cancer, and non-small cell lung cancer [186]. The POMotors were constructed by conjugating peroxidase-like P_2_W_18_Fe_4_ polyoxometalates (POMs) with PDA, enabling NIR light-driven photothermal propulsion. The combination of NIR-triggered thermophoresis and anti-EGFR targeting significantly enhanced tumor penetration and cellular internalization in vivo, thereby substantially increasing drug accumulation within the tumor tissue. Similarly, we previously reported the use of the targeting molecule cyclic RGD (cRGD) to enhance the accumulation of photothermal nanomotors at tumor sites (Figure 12b) [146]. cRGD, a cyclic tripeptide composed of arginine, glycine, and aspartic acid (Arg-Gly-Asp), exhibits high specificity for binding to integrin receptors such as αvβ3 and αvβ5. These receptors are highly expressed on the surface of many tumor cells [189]. Therefore, the selection of targeting molecules should be tailored to the specific cancer type, as different cancer cells overexpress distinct receptors.

Macrophages possess a diverse array of cytokine receptors on their membrane surfaces, endowing them with inherent inflammatory targeting capabilities [192]. This characteristic is frequently leveraged for applications in both cancer and inflammation targeting [193]. For instance, Luo et al. developed Janus nanomotors coated with M2 macrophage membranes (Motor@M2M) for inflammation targeting (Figure 12c) [190]. After coating with the M2M membrane (yielding Motor@M2M), the nanomotors were further encapsulated within a sodium alginate gel (SAM) using microfluidic technology, resulting in Motor@M2M@SAM. The M2M coating enhances the MNMs’ targeting accuracy towards inflammatory tissues and also functions as a decoy to neutralize inflammatory cytokines. In an oxidative stress environment, the MnO_2_ component catalyzes the decomposition of H_2_O_2_. This reaction generates O_2_ bubbles which propel Motor@M2M, facilitating penetration across the mucus barrier into inflamed colon tissue. In addition to immune cell membrane-based targeting for immune recognition, the homologous targeting afforded by tumor cell membranes represents another common strategy for enhancing the targeting of nanomaterials in cancer therapy [194]. Illustrating this, Wang et al. camouflaged Janus Pt-Au nanospheres (JPGSs) with tumor cell membranes, creating a nanomotor (4T1-JPGSs-IND) with homotypic targeting capability towards cancer cells for photothermal tumor therapy (Figure 12d) [191]. Under NIR irradiation, the combined effect of self-thermophoretic propulsion and homologous targeting enables 4T1-JPGSs-IND to penetrate deeply into tumor tissue. This innovative design enhances the efficacy of PTT while reducing side effects, thereby improving overall cancer treatment outcomes.

### 3.2. Tumor Penetration of MNMs

**Figure 13:**
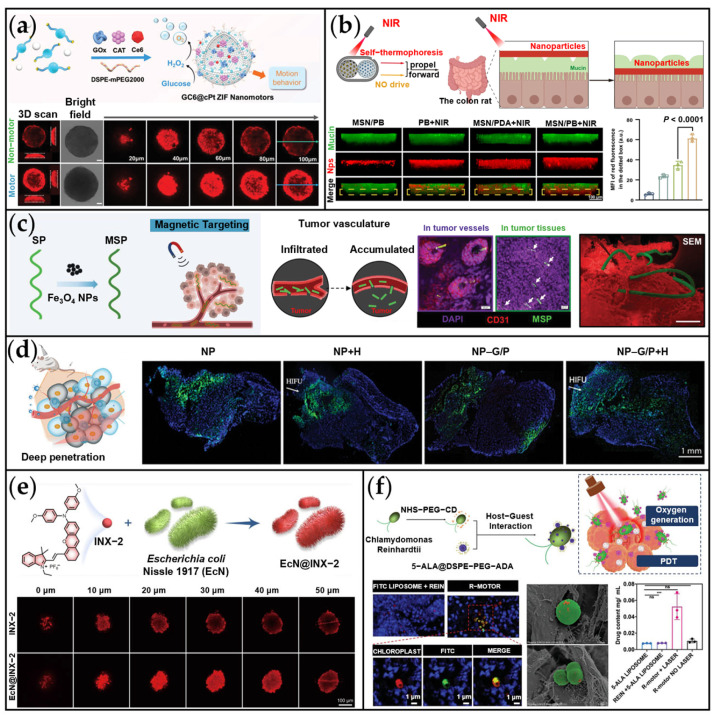
Tumor penetration of MNMs. (**a**) Schematic illustrating the self-propulsion mechanism of GC6@cPt ZIF nanomotors (up) and corresponding CLSM images showing their penetration into 3D multicellular tumor spheroids (MTSs) (down). Scale bar = 50 µm (reproduced from [97] with permission from John Wiley and Sons). (**b**) Mechanism of MPBC movement direction under NIR irradiation (top) and CLSM images demonstrating nanoparticle penetration across the intestinal mucus barrier (bottom). Scale bar = 100 µm (reproduced from [148] with permission from Springer Nature). (**c**) Schematic design of magnetic *S. platensis*-based microswimmers for tumor targeting (left) and their infiltration and accumulation mechanism within tumor tissue via tumor vasculature (right) (reproduced from [159] with permission from John Wiley and Sons). (**d**) Immunofluorescence images of tumor sections depicting the accumulation and penetration profiles of nanomotors post-injection. Scale bar = 1 mm (reproduced from [160] with permission from John Wiley and Sons). (**e**) CLSM images of EcN@INX-2 nanomotors and INX-2 penetrating CT26 tumor spheroids (reproduced from [166] with permission from Springer Nature). (**f**) Mechanism of R-motor for targeted tumor accumulation, in situ oxygen generation, and subsequent PDT, *C. reinhardtii* labeled in green and liposomes labeled in red. Data were statistically analyzed using one-way ANOVA. *** *p* ≤ 0.001, n.s. non-significant. Scale bar = 1 μm. (reproduced from [167] with permission from John Wiley and Sons).

The dense extracellular matrix (ECM) and associated fibroblasts constitute a tumor stromal barrier that severely impedes nanomedicine diffusion and penetration within the TME, significantly diminishing therapeutic efficacy [195,196]. Consequently, overcoming this tumor interstitial barrier is paramount for improving tumor treatment outcomes. MNMs offer a promising solution due to their intrinsic autonomous propulsion and tumor-targeting capabilities. Under applied driving forces, MNMs can effectively penetrate the dense ECM and access deeper tumor regions, thereby enhancing intertumoral drug accumulation and therapeutic impact [197,198]. To demonstrate this potential, we engineered a bubble-propelled nanomotor based on a zeolite imidazole framework (ZIF) coated with Pt nanoparticles (GC@cPt ZIF) (Figure 13a) [97]. This platform integrates GOx, CAT, and cisplatin within the ZIF structure. Through an enzyme-catalyzed cascade reaction, the nanomotor achieves self-propulsion via O_2_ bubble generation, simultaneously alleviating tumor hypoxia. In a 3D multicellular tumor spheroid model, the GC@cPt ZIF nanomotors exhibited significantly deeper penetration compared to passive control particles. Notably, clear red fluorescence signal (indicating nanomotor presence) was detectable at depths up to 100 µm, contrasting with the non-motor group (Figure 13a). Furthermore, both penetration depth and average fluorescence intensity for the nanomotors were substantially higher than those of the controls. This study underscores the significant potential of enzymatically powered nanomotors in overcoming the critical challenge of the tumor ECM barrier, facilitating deeper drug delivery, and advancing synergistic cancer therapies.

For physically driven MNMs, Wang et al. reported that orally administered CMPCB nanomotors utilized dual propulsion mechanisms: self-thermophoresis and NO bubble generation [148]. This combination enabled CMPCB to rapidly penetrate the intestinal mucus barrier, significantly enhancing therapeutic efficacy against colorectal cancer (Figure 13b). Under NIR irradiation, the nanomotor actively traversed the barrier, resulting in fluorescence intensities in the lower mucus layer that were 1.8-, 2.6-, and 10.0-fold higher than those in the control groups, respectively. These dual-driven bioactive nanomotors were specifically engineered to overcome both the intestinal mucus and epithelial barriers efficiently. The core innovation lies in the reversible modulation of the epithelial barrier combined with autonomous movement to augment drug delivery, presenting a novel paradigm for developing oral anticancer drugs. As depicted in Figure 13c, the magnetic Spirulina biohybrid nanophoresis system (MSP) demonstrates effective enrichment at tumor sites under an externally applied magnetic field [159]. Guided magnetically, the MSP navigated across the tumor vessel wall and was observed localizing laterally within the tumor vasculature. Moreover, MSP accumulation was detected in deeper regions of the tumor tissue. Overall, this study demonstrated that magnetic navigation enables these biohybrid nanoalgae to penetrate the tumor vasculature and achieve drug accumulation deep within the tumor. Nanomotors propelled by high-intensity focused ultrasound (HIFU) also exhibit significant tumor penetration capabilities. For instance, the HIFU-driven NP-G/P nanomotor developed by Yu et al. achieved a remarkable penetration depth within tumors (Figure 13d) [160]. Under HIFU propulsion, nanoparticles in the NP-G/P group aligned with the direction of irradiation and penetrated the tumor tissue to a depth of several millimeters.

Microbial tropism can significantly enhance the motor’s tumor penetration efficiency. As previously discussed, the hypoxic targeting capability of EcN@INX-2, based on the facultative anaerobic characteristics of EcN, enables specific targeting and penetration into hypoxic tumor regions (Figure 13e) [166]. LSM imaging confirmed deep penetration of EcN@INX-2 throughout the tumor spheroid, whereas free INX-2 remained predominantly confined to the surface layer. Notably, EcN@INX-2 increased intratumoral fluorescence intensity by a factor of three compared to free INX-2. This biomimetic system leverages EcN’s hypoxic tropism to enhance INX-2 accumulation at tumor sites, enabling the integration of multimodal photothermal therapy with immunotherapy and thereby improving therapeutic efficacy against colon cancer. Similarly, phototactic algae can be utilized for tumor targeting. By irradiating the tumor region with NIR light, it is possible to promote the enrichment of phototactic algae in the target area. For instance, the aforementioned biohybrid micromotor (R-motor) employs natural *C. reinhardtii* for targeted PDT (Figure 13f) [167]. This system exploits the algae’s inherent phototaxis to achieve light-guided tumor targeting under 680 nm laser irradiation. SEM analysis of tumor sections after 30 min of light exposure revealed red chloroplast fluorescence deep within the tumor, confirming successful phototaxis-driven penetration. Furthermore, the drug concentration achieved within the tumor was approximately fivefold higher than that observed in the control group.

## 4. Synergistic Treatment of MNMs with Other Therapies

Self-propelled MNMs exhibit exceptional tumor-targeting capabilities and motility, enabling their substantial accumulation within tumor tissues. This unique property provides a promising platform for advancing cancer therapy, as it facilitates highly precise tumor targeting while minimizing damage to healthy tissues. Furthermore, MNMs serve as an outstanding integrated platform for the combination of multiple therapeutic modalities. Through synergistic effects, this approach maximizes tumor eradication. This section specifically outlines current strategies for utilizing MNMs in combination therapy and provides an in-depth exploration of their underlying synergistic mechanisms.

### 4.1. Chemodynamic Therapyc (CDT)

**Figure 14:**
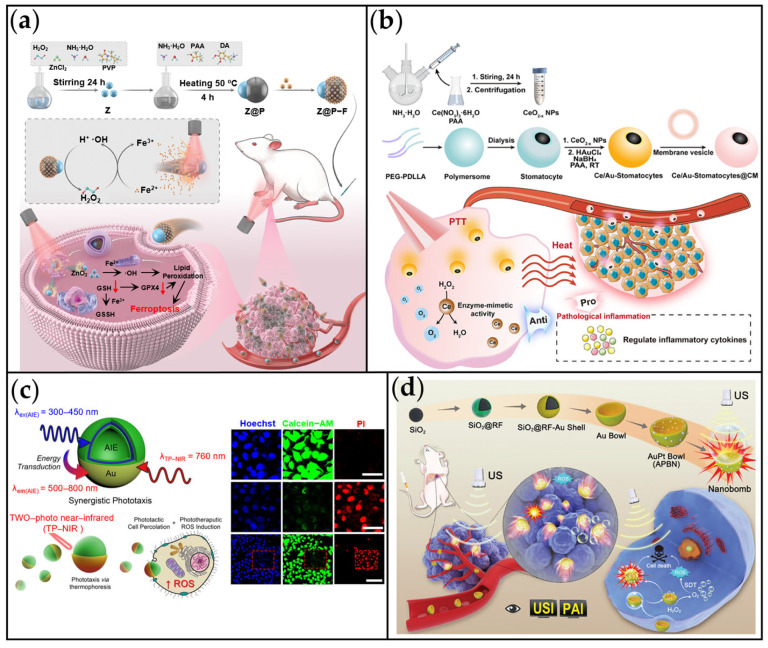
Synergistic treatment of MNMs with other therapies. (**a**) Schematic of self-thermophoretic nanomotors enabling acidic microenvironment-triggered ROS supply for enhanced tumor CDT/PTT (reproduced from [201] with permission from John Wiley and Sons). (**b**) Preparation of Ce/Au-Stomatocytes@CM for motion-promoted and inflammation-alleviated PTT of tumors (reproduced from [202] with permission from John Wiley and Sons). (**c**) TP-NIR activation of nanomotors triggers dual behaviors: enhancing cellular interactions/uptake alongside phototherapeutic ROS generation for localized cell toxicity (reproduced from [203] with permission from Springer Nature). (**d**) Schematic of the synthesis process of APBN sonosensitizers as nanobombs, and their USI/PAI-guided deep orthotopic liver tumor SDT (reproduced from [204] with permission from John Wiley and Sons).

CDT, a cancer therapeutic strategy, leverages Fenton or Fenton-like reactions to generate cytotoxic •OH radicals within the TME, thereby inducing tumor cell death [199]. However, the efficacy of CDT is often limited by the insufficient concentration of endogenous H_2_O_2_ in the TME [200]. To overcome this constraint, Hu et al. developed an intelligent nanoplatform: a NIR laser-driven, self-thermophoretic nanomotor (Z@P-F) capable of generating H_2_O_2_ to augment Fe^2+^-mediated CDT (Figure 14a) [201]. The Z@P-F nanomotor comprised a ZnO_2_ core coated asymmetrically with Fe^2+^-incorporated PDA. In the acidic TME, the ZnO_2_ core decomposes continuously, supplying H_2_O_2_ (ZnO_2_ + 2H^+^ → H_2_O_2_). Under NIR irradiation, the asymmetric PDA coating generates localized heat, enabling self-thermophoretic propulsion at speeds up to 5.08 μm/s. This active motion significantly enhances tumor penetration and facilitates lysosomal escape. This significantly enhanced tumor penetration and lysosomal escape. Subsequently, the released Fe^2+^ ions react with the self-generated H_2_O_2_ via the Fenton reaction, amplifying ROS production. This synergistic action disrupts mitochondrial function, depletes glutathione, and induces lipid peroxidation, ultimately triggering ferroptosis. This work presents a multifunctional nanoplatform combining active self-propelled motion, controlled ROS generation, and PTT for effective solid tumor treatment.

### 4.2. Photothermal Therapy (PTT)

PTT utilizes the photothermal effect of photothermal agents (PTAs) to convert light energy into heat. This localized heating elevates tumor and surrounding microenvironment temperatures to 60–80 °C, inducing cancer cell apoptosis or necrosis [205]. PTT offers distinct advantages over other therapeutic modalities. First, externally tunable laser irradiation enables precise tumor targeting. Additionally, PTT is a highly effective, non-invasive treatment capable of eradicating various malignancies [206]. However, several challenges limit PTT’s therapeutic efficacy. Effective delivery of PTAs to tumor sites remains a significant hurdle. Furthermore, PTT can cause inflammatory damage to adjacent normal tissues, and achieving deep tumor penetration while minimizing such damage remains exceptionally challenging. The emergence of MNMs offers a promising solution. Harnessing their photothermal properties, MNMs facilitate deeper tumor penetration and substantially reduce side effects through light-controlled targeted delivery. Guo et al. developed an oxygen/photothermal dual-driven stomatocyte nanomotor (Ce/Au-Stomatocytes@CM) to enhance breast tumor PTT efficacy while concurrently mitigating PTT-induced inflammation (Figure 14b) [202]. These nanomotors comprise Au NPs and catalase-mimetic cerium oxide nanoparticles (CeO2-x NPs) encapsulated within biodegradable PEG-PDLLA stomatocytes, further cloaked with cancer cell membranes (CCM) for homotypic tumor targeting. The asymmetric stomatocyte structure enables autonomous motion via two distinct mechanisms: (1) O_2_-mediated propulsion generated by tumor H_2_O_2_ decomposition catalyzed by CeO_2-x_ NPs; and (2) NIR (660 nm)-activated thermophoretic motion driven by Au NPs, facilitating directional penetration. Crucially, CeO_2-x_ NPs also effectively mitigate PTT-triggered inflammation by scavenging ROS and significantly suppressing proinflammatory cytokine production. This study thus introduces a multifunctional nanoplatform that integrates autonomous propulsion, hypoxia modulation, targeted PTT, and anti-inflammatory action, presenting a highly promising strategy for advancing inflammation-mediated precision oncology.

### 4.3. Photodynamic Therapy (PDT)

PDT has emerged as a promising non-invasive approach for cancer treatment in recent years, demonstrating distinct advantages [207]. In therapeutic applications, photosensitizers (PSs) accumulate near tumor cells and, upon irradiation with light of specific wavelengths, are activated to produce ROS, including singlet oxygen (^1^O_2_) and superoxide anion radicals (O_2_^−•^). These ROS primarily induce tumor cell death through apoptosis or necrosis [208]. Cao et al. developed a hybrid nanomotor system designed to enhance PDT under NIR light activation, integrating aggregation-induced emission (AIE) polymersomes with asymmetric Au nanoshells (Figure 14c) [203]. Specifically, they covalently grafted second-generation AIE motifs (TPEDC) onto biodegradable PEG-PTMC block copolymers, leading to self-assembled vesicles exhibiting stable fluorescence and high two-photon absorption efficiency (λex(AIE) = 300–450 nm). The asymmetric Au nanoshell coating enabled NIR-driven thermophoretic propulsion (reaching speeds up to 8.5 μm/s) via plasmonic heating. Crucially, the AIE components generated ROS upon two-photon excitation at 760 nm. This synergy between AIE-mediated ROS generation and Au-enhanced directional motility resulted in rapid, localized cytotoxicity with precise spatial control as NIR irradiation for 80 s induced membrane permeabilization, while 48 s of irradiation triggered significant intracellular ROS production, achieving targeted single-cell ablation. In vitro experiments on HeLa cells confirmed this spatially controllable cytotoxicity under NIR irradiation, with ROS-mediated apoptosis (marked by red fluorescence) observable within 80 s. This work establishes a fuel-free, dual-functional platform combining phototaxis and phototherapy. By harnessing energy-transducing AIE-Au interactions for directional motion, it overcomes the limitations inherent in Brownian diffusion, offering a promising strategy for precision oncology applications.

### 4.4. Sonodynamic Therapy (SDT)

SDT represents an innovative modality for cancer treatment. It employ US to activate sonosensitizers, which subsequently react with O_2_, generating substantial ROS to effectively eradicate malignant tumors [209]. Consequently, addressing tumor hypoxia is crucial for enhancing SDT efficacy. Furthermore, the integration of sonosensitizers with MNMs facilitates ultrasound-mediated delivery of these agents into the tumor interior, thereby potentiating the therapeutic outcome of SDT. To overcome the limitations of SDT for orthotopic liver cancer, Lin et al. developed US-activated Au-Pt bowl-shaped nanobombs (APBNs) as multifunctional sonosensitizers (Figure 14d) [204]. APBNs feature a specifically engineered concave structure designed to alleviate tumor hypoxia, enhance cavitation effects, and improve intratumoral penetration. Leveraging Pt’s catalase-like activity, APBNs catalytically convert endogenous H_2_O_2_ into O_2_ bubbles. These bubbles accumulate within the bowl-shaped grooves, serving as stabilized cavitation nuclei. Under US irradiation, these stabilized bubbles undergo vigorous oscillations, amplifying mechanical damage, while concurrently generating ROS for synergistic tumor destruction. Furthermore, the asymmetric design of APBNs enables US-propelled motion, enhancing penetration into dense tumor tissues. Dual-modality ultrasound/photoacoustic imaging enabled precise tumor targeting. Specifically, photoacoustic imaging capitalized on the strong NIR absorption properties of APBNs to visualize deep tumors. This work presents an innovative strategy integrating catalytic O_2_ generation, cavitation-enhanced SDT, and US-driven mobility, offering a promising theranostic platform for treating deep-seated tumors.

### 4.5. Gas Therapy (GT)

**Figure 15:**
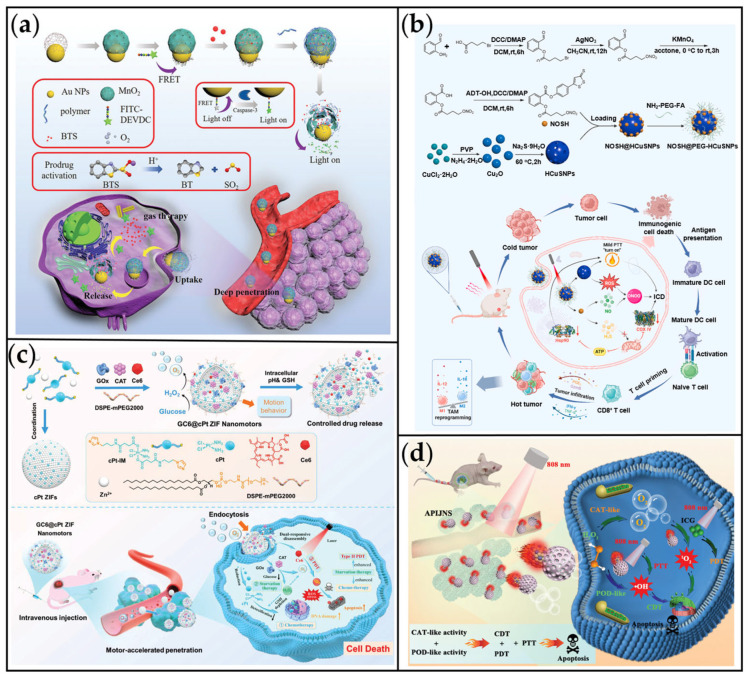
Synergistic treatment of MNMs with other therapies. (**a**) Preparation of BTS-Au@MnO_2_ nanomotors and self-propelled gas therapy (reproduced from [79] with permission from John Wiley and Sons). (**b**) Construction process of NOSH@PEG-HCuSNPs and schematic of their NIR-triggered, programmable cascading oncotherapy via mPTT/gas/ROS-reinforced ICD (reproduced from [218] with permission from Elsevier). (**c**) Tailoring enzyme-engineered cisplatin prodrug nanomotors for synergistic breast cancer therapy (reproduced from [97] with permission from John Wiley and Sons). (**d**) Schematic illustration of parachute-like APIJNSs for synergistic CDT/PTT/PDT (reproduced from [219] with permission from John Wiley and Sons).

Gas therapy (GT), which utilizes therapeutic gaseous molecules, represents a promising approach for treating various diseases, including wound healing, inflammatory conditions, cardiovascular disorders, and cancers. In tumor therapy specifically, elevated concentrations of gaseous signaling molecules—such as NO, H_2_S, CO, and H_2_—demonstrate cytotoxic effects against cancer cells [210,211,212,213]. These molecules, crucial for maintaining physiological homeostasis as endogenous messengers, exhibit minimal side effects on normal cells or tissues. Recent studies further indicate that GT synergizes effectively with other treatment modalities to enhance anticancer efficacy [214,215,216,217]. To address limitations in deep-tissue GT delivery, Yue et al. engineered an asymmetrical, self-propelled Au@MnO_2_ nanomotor capable of enhancing GT efficacy in deep tumor regions while enabling real-time therapeutic monitoring (Figure 15a) [79]. The nanomotor leverages catalytic decomposition of endogenous H_2_O_2_ into O_2_ by its MnO_2_ shell for propulsion, facilitating deep tumor penetration. The hollow MnO2 structure encapsulates a SO_2_ prodrug (benzothiazole sulfinate, BTS), which releases therapeutic SO_2_ in response to the TME, inducing oxidative stress and apoptosis. Concurrently, a caspase-3-responsive peptide (DEVD) conjugated to FITC on the Au core enables “self-reporting” of apoptosis: fluorescence remains quenched via Förster resonance energy transfer (FRET) until caspase-3 cleavage during apoptosis activates the signal. In vitro and in vivo studies demonstrated efficient tumor penetration, selective SO_2_ release in cancer cells (versus normal cells), and significant tumor suppression—achieving a 70% reduction in B16 melanoma models—with minimal systemic toxicity. The nanomotor’s asymmetrical design conferred superior directional motility and tissue penetration compared to symmetrical SiO_2_@MnO_2_ counterparts. This integrated platform, combining targeted gas delivery, self-propulsion, and real-time therapeutic feedback, collectively represents a promising strategy to overcome translational barriers in GT and advance precision cancer treatment.

### 4.6. Immunotherapy (IT)

Immunotherapy (IT) represents a highly promising strategy for achieving cancer remission by inducing a systemic and sustained immune response that promotes tumor regression and suppresses metastasis [220,221,222,223,224]. However, the inherent low immunogenicity of tumor cells often enables immune evasion [214,215,216,217,218]. While inducing apoptosis via anticancer agents remains a key therapeutic approach, the subsequent phagocytosis of apoptotic tumor cells by macrophages contributes to immunosuppression within the TME and impedes host immune system activation. Consequently, effectively stimulating anti-tumor immunity requires the activation of alternative ICD pathways to enhance tumor immunogenicity.

NO is a prototypical activator of ICD pathways. Within tumor cells, NO induces the production of peroxynitrite anions (ONOO^−^) [225,226], which promotes macrophage polarization from the immunosuppressive M2 phenotype towards the pro-inflammatory M1 phenotype, thereby reversing TME immunosuppression and enhancing anti-tumor responses. Zhang et al. developed a NIR-triggered programmable nanomotor (NOSH@PEG-HCuSNPs) that synergistically integrates H_2_S and NO gas therapies with mild photothermal therapy (mPTT) to achieve enhanced antitumor efficacy via ICD induction (Figure 15b) [218]. This nanomotor was constructed by encapsulating the dual gas donor NOSH (which releases both H_2_S and NO) within hollow mesoporous copper sulfide nanoparticles (HCuSNPs), surface-modified with PEG-folic acid for improved tumor targeting. Upon 808 nm laser irradiation, the nanomotor simultaneously achieves localized mPTT (maintained at 45 °C) and triggers the release of H_2_S and NO. The liberated H_2_S inhibits cytochrome c oxidase subunit IV (COX IV), inducing mitochondrial dysfunction and ATP depletion. This ATP depletion, in turn, suppresses heat shock protein 90 (HSP90) expression, sensitizing the tumor cells to thermal ablation. Concurrently, the released NO reacts with photothermally generated ROS, generating cytotoxic ONOO^−^ and amplifying oxidative stress. This synergistic strategy combining mPTT and GT effectively enhanced ICD, as evidenced by an increased number of mature dendritic cells (DCs), promotion of M1 macrophage polarization, activation of cytotoxic T lymphocytes (CTLs), sensitization of systemic immunity, and inhibition of distant tumor growth. This work establishes a non-invasive, immunologically amplified approach with significant translational potential for the treatment of metastatic cancer.

### 4.7. Multiple Modality Therapy

We developed glucose-fueled enzymatic nanomotors (GC6@cPt ZIFs) based on cisplatin-prodrug-derived zeolitic imidazolate frameworks (ZIFs) for synergistic multimodal cancer therapy (Figure 15c) [97]. These nanomotors incorporated GOx, CAT, and the photosensitizer chlorin e6 (Ce6) within the cisplatin-ZIF framework. Enzymatic cascade reactions drove self-propulsion via O_2_ bubble propulsion, enhancing tumor penetration and accumulation. Within the TME—characterized by acidic pH and elevated glutathione levels—the ZIFs disintegrated, releasing cisplatin, Ce6, and the enzymes. Concurrently, O_2_ generation alleviated hypoxia, augmenting Ce6-mediated PDT, while glucose depletion enabled starvation therapy. In vitro and in vivo studies demonstrated deep tumor penetration (up to 100 µm), amplified oxidative stress (via elevated ROS and depleted GSH), and synergistic antitumor effects (91.4% apoptosis in vitro; 80% survival rate in vivo). By integrating chemotherapy, PDT, and metabolic disruption, these nanomotors provide a robust platform to overcome TME limitations and enhance therapeutic efficacy.

Zhang et al. presented a dual-source-driven, parachute-like Janus nanomotor (APIJNS) for enhanced tumor penetration and synergistic cancer therapy combining PTT, PDT, and CDT (Figure 15d) [219]. APIJNS enabled collaborative treatment through: (1) Dual Propulsion: Achieving velocities up to 135.36 µm/s via NIR-induced self-thermophoresis and H_2_O_2_-catalyzed bubble thrust, significantly enhancing tumor penetration; (2) Multimodal Catalysis: Au_2_Pt nanoparticles exhibited POD-like activity (generating •OH for enhanced CDT) and CAT-like activity (producing O_2_ to alleviate hypoxia and enable indocyanine green (ICG)-mediated ^1^O_2_ generation under NIR irradiation for PDT); (3) Potent Photothermal Conversion: High efficiency (36.23%) under 808 nm laser irradiation induced localized hyperthermia (ΔT ≈ 43 °C), amplifying ROS generation and enhancing PTT. This dual propulsion system coupled with triple therapeutic synergy (PTT/PDT/CDT) overcomes limitations of passive diffusion and tumor hypoxia, offering an innovative strategy for active nanomotor-mediated tumor targeting and microenvironment modulation to enhance cancer therapy.

## 5. Application of MNMs in Tumor Diagnostics

**Figure 16:**
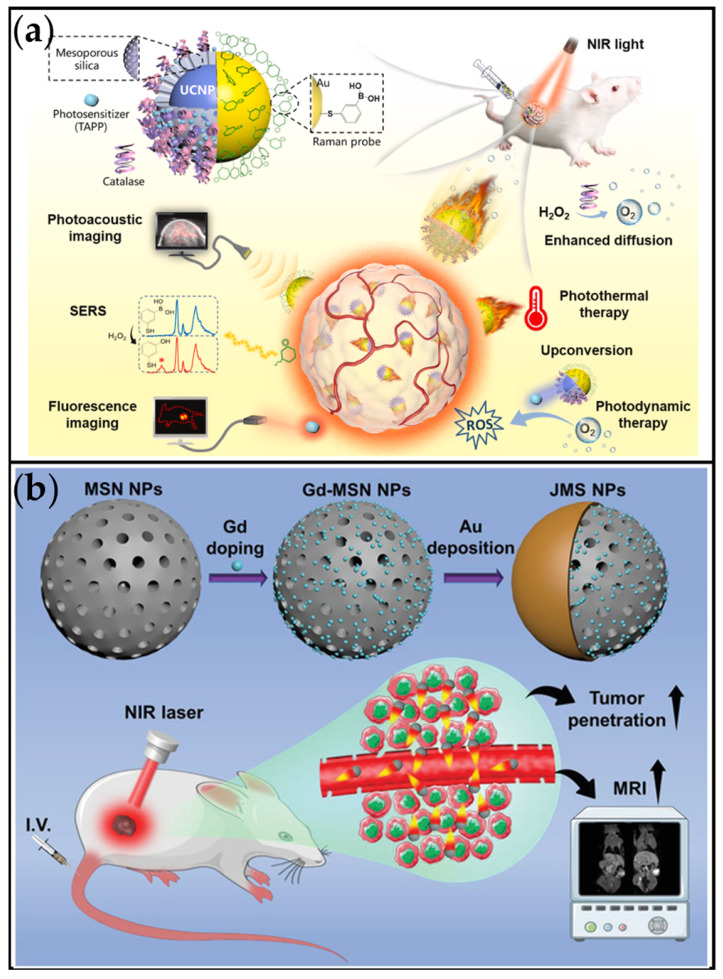
Application of MNMs in Tumor Diagnostics. (**a**) Schematic illustrating the dual-source powered nanomotor for surface-enhanced Raman scattering (SERS) biosensing and multimodal cancer photo-theranostics (reproduced from [145] with permission from John Wiley and Sons). (**b**) Schematic illustration of preparation process of JMS nanomotors and the NIR-powered JMS nanomotors for tumor deep penetration and enhanced MRI (reproduced from [227] with permission from Elsevier).

Nanomedicine holds exceptional potential for cancer diagnosis and treatment. Specifically engineered MNMs can function as contrast agents, enhancing the sensitivity and resolution of tumor detection with advanced imaging techniques [145,166,227,228]. Beyond detecting tumor markers, these imaging agents enable precise visualization of tumor tissues. This capability facilitates not only accurate cancer diagnosis but also precise image-guided therapy [228]. To date, diverse phototriggered and magnetically triggered bioimaging and therapeutic agents—characterized by noninvasiveness, remote controllability, and high efficiency—have significantly advanced biomedical applications [223]. For instance, the development of advanced imaging techniques such as photoacoustic imaging (PAI), fluorescence imaging (FLI), and magnetic resonance imaging (MRI) has substantially enhanced the cancer diagnostic capabilities of MNMs [229]. Chen et al. developed a dual-source-driven Janus nanomotor that integrates surface-enhanced Raman scattering (SERS) sensing, FLI, and PAI for enhanced tumor imaging while simultaneously enabling PDT and PTT (Figure 16a) [145]. This nanomotor combines upconversion nanoparticles (UCNPs), a mesoporous silica layer, a Au nanoshell, and multiple surface modifications—including the photosensitizer TAPP, Raman reporter 3-MPBA, and CAT—to achieve synergistic multifunctionality. Self-propulsion, powered by catalase-mediated H_2_O_2_ decomposition and NIR-induced self-thermophoresis, facilitates deep tumor penetration. Within the TME, H_2_O_2_ oxidizes 3-MPBA to 3-HTP, yielding a Raman peak intensity that linearly correlates with H_2_O_2_ concentration (100 nM to 10 μM), thereby enabling quantification. TAPP’s fluorescence allows tumor labeling and fluorescence imaging, while the Au nanoshell significantly enhances the PAI signal in tumor regions. Therapeutically, UCNPs convert visible light to activate TAPP for ^1^O_2_ generation. Simultaneously, the Au nanoshell irradiated at 808 nm maintains a temperature of 42 °C, meeting tumor PTT requirements. This work presents an intelligent integrated nanoplatform for precision cancer therapy, combining imaging, detection, and multimodal treatment within a unified framework.

Zheng et al. developed a NIR-activated Janus mesoporous silica nanomotor (JMS nanomotor) to enhance MRI of solid tumors (Figure 16b) [227]. The JMS nanomotors comprised a Gd-doped mesoporous silica core, serving as an MRI contrast agent, and an asymmetric hemispherical Au coating. This gold layer enabled NIR-responsive propulsion via localized thermophoresis. Upon NIR irradiation (1.5–2.5 W/cm^2^), the asymmetric Au layer generated a thermal gradient, propelling the nanomotors at velocities reaching 3.49 µm/s. In vitro studies demonstrated that NIR-driven propulsion significantly enhanced tumor cell membrane penetration, resulting in a 4.8-fold increase in cellular uptake and consequently boosting T1-weighted MRI signal intensity. In vivo investigations using 4T1 tumor-bearing mice revealed that NIR activation facilitated deeper tumor penetration and accumulation, achieving a 1.73-fold enhancement in MRI contrast within the tumor tissue. This study represents a pioneering advancement in integrating active nanomotor propulsion with real-time MRI tracking, offering a robust platform for precise tumor diagnosis and theranostic applications.

## 6. Conclusions and Outlook

The emergence of MNMs has transformed cancer diagnosis and therapy, offering unparalleled solutions to the limitations of conventional oncology treatments. By harnessing autonomous motion, targeted drug delivery, and synergistic therapeutic approaches, MNMs demonstrate substantial potential to enhance tumor penetration, improve therapeutic efficacy, and reduce systemic toxicity. This paper comprehensively reviews MNMs propulsion mechanisms—including chemical, external energy field (e.g., magnetic, acoustic, optical), and biological drives—alongside their applications in addressing challenges posed by the TME. We summarize representative MNMs designs, detailing structures, propulsion mechanisms, and velocities (Table 1 and Table 2). Targeting strategies, surface modifications, and therapeutic modalities are systematically categorized (Table 3 and Table 4). These syntheses aim to advance understanding of MNMs development and inform the design of more effective oncology platforms. For future clinical prospects, we anticipate MNMs will fully realize their biomedical potential by integrating diagnostics and therapy (“theranostics”) in cancer trials. However, critical challenges must be addressed:

(1) **Biocompatibility and toxicity issues.** Most of the reported nanomaterials are composed of inorganic materials. Although most of these materials are considered biocompatible and biodegradable, they may still exhibit strong immunogenicity and have limitations regarding the maximum tolerated dose. In addition, the degradation rates of these materials and their in vivo metabolic pathways remain unclear and require further investigation to confirm their long-term biological safety.
(2) **Mechanical energy conversion efficiency.** In complex physiological environments (e.g., bloodstream dynamics), MNMs must overcome fluid viscosity and flow velocity. Higher propulsion power translates to greater obstacle-surmounting capacity and task efficiency. However, fuel molecule concentrations in blood or the TME are often limited, necessitating MNMs with enhanced energy conversion efficiency and power output.
(3) **Targeting precision and biological barrier penetration.** Overcoming barriers like the blood–brain barrier, intestinal mucus, and tumor stroma remains formidable. Future strategies should emphasize: (i) molecular targeting (e.g., engineered antibodies, specific peptides) and (ii) physical field guidance (e.g., magnetic fields, near-infrared light, ultrasound) to improve accuracy and penetration.
(4) **Image-guided therapy limitations.** Current MNMs imaging techniques (FLI/MRI/PAI) enable localized tumor imaging but lack real-time monitoring depth. Integrating multimodal imaging and enabling real-time tracking are essential for treatment progress assessment, precise localization, and safety.
(5) **Clinical translation hurdles.** Current MNM-based cancer research remains confined to cellular and animal studies. Clinical data are scarce, and standardized human efficacy/toxicity evaluation frameworks are lacking. Batch-to-batch variations during preparation further impede clinical translation.

In conclusion, MNMs represent a transformative oncology paradigm, integrating nanotechnology and biomedicine to enable precision therapy. Despite challenges in biocompatibility, scalability, and clinical validation, interdisciplinary innovation offers promising pathways to unlock their full potential. Future research must prioritize translational feasibility to accelerate the transition of these dynamic nanoscale systems from bench to bedside, ultimately improving outcomes for cancer patients globally.

## Figures and Tables

**Table 1:** Comparison of substrate, propulsion mechanism, and composition of chemically driven MNMs.

Substrate	Structure and Components	Propulsion Mechanisms	Speed (μm/s) or Diffusion Coefficient (D_e_, μm^2^/s)	Ref.
H_2_O_2_	Polydimethylsiloxane (PDMS) plate with Pt-plated porous glass.	O_2_ bubble propulsion 2H_2_O_2_ → O_2_ ↑ + 2H_2_O	1–2 cm/s	[86]
	Bilayer polyaniline/Pt microtubes.		~120 μm/s	[87]
	Reduced nanographene oxide (n-rGO)/Pt microtubes.		~246 µm/s	[88]
	Poly(ethylene glycol)-b-polystyrene (PEG-b-PS) bowl-shaped stomatocytes/PtNPs.		~35 μm/s	[89]
	Cruciate flower-like zeolitic imidazolate framework-L (ZIF-L) incorporating β-lactoglobulin and catalase.		1.27~2.65 μm^2^/s in 0.3–3% H_2_O_2_ at pH 7.0, 0.65–0.81 μm^2^/s in 0.3–3% H_2_O_2_ at pH 5.0.	[90]
	Janus TiO_2_ core-mesoporous organosilica (PMO) rods modified with natural CATs.	Asymmetric ionic concentration gradient-based self-diffusiophoresis H_2_O_2_ → 2H^+^ + 2e^−^+ O_2_ 2H^+^ + 2e^−^ + H_2_O_2_ → 2H_2_O	18.71 μm/s, 4.60 μm^2^/s in 200 μM H_2_O_2_	[91]
	Asymmetric and hollow-open Au-silica tadpole-shaped MNMs (AHOASTs).	Asymmetric O_2_ concentration gradient-based self-diffusiophoresis 2H_2_O_2_ → O_2_ + 2H_2_O	3.1 μm^2^/s in 1.5% H_2_O_2_	[92]
	Janus Au nanorod-Pt nanomotor (JAuNR-Pt)	H^+^ and O_2_ gradient-based self-electrophoresis H_2_O_2_ → 2H^+^ + 2e^−^ + O_2_ 2H^+^ + 2e^−^ + H_2_O_2_ → 2 H_2_O	10.18 μm/s, 2.38 μm^2^/s	[41]
Glucose	Cationic Au nanoclusters adsorb GOx and CAT (CAuNCs@HA)	O_2_ bubble propulsion Glucose + O_2_ →H_2_O_2_ + Gluconic acid 2H_2_O_2_ → O_2_ ↑ + 2H_2_O	25.25 ± 0.33 μm/s	[96]
	Cisplatin (cPt) prodrug forms ZIFs and encapsulates GOx and CAT.		2 μm/s in 10 mM glucose solution and 1.4 μm/s in 5 mM glucose solution	[97]
	Nanoparticles formed the by cross-linking of GOx and ferritin.	Self-diffusiophoresis Glucose + O_2_ →H_2_O_2_ + Gluconic acid	3.07 μm/s in 100 mM glucose solution	[98]
	Carbonaceous nanoflask encapsulates GOx and CAT (CNF)		Hydrophilic L-CNF motor: 0.97 μm/s. Hydrophobic B-CNF motor: 0.82 μm/s	[100]
	Anisotropic hollow multishell structures incorporating Au/Pt nanoparticles.		7.31 μm/s, 4.96 μm^2^/s	[99]
Arginine	Arginine binds to HPAM via electrostatic interactions to form composite nanoparticles.	NO bubble propulsion Arginine + ROS → NO ↑ + Citrulline	3~13 μm/s in 20% H_2_O_2_ solution	[78]
	Heparin/folic acid binds to arginine through electrostatic interactions to form composite nanoparticles.		N/A	[110]
	The poly(N-vinylcaprolactam) (PVCL)-based nanogel co-loads arginine through hydrophobic/electrostatic interactions.		4–11 μm/s in 1–5% H_2_O_2_ solution	[89]
	Polymerization of an arginine derivative with a diselenide crosslinker (Arg-Me).		5.2 ± 1.0 μm/s in the tumor cell environment	[108]
Glucose and arginine	Janus dendritic mesoporous silica/Au nanoparticles encapsulating arginine.	Self-diffusiophoresis Glucose + O_2_ → H_2_O_2_ + Gluconic acid Arginine + H_2_O_2_ → NO ↑ + Citrulline	10.9 μm/s in 1% glucose solution	[112]
Urea	Asymmetrically immobilizing urease on natural platelets.	Self-diffusiophoresis Urea + H_2_O → CO_2_ + 2NH_3_ CO_2_ and NH_3_ + H_2_O → CO_3_^2−^ + NH_4_^+^	~7 μm/s in 200 mM urea and De 2~3 μm^2^/s in simulated urine	[52]
	Janus Au nanoparticles functionalized with urease (UPJNMs).		The diffusion coefficient (D_e_) of UPJNMs increase as their size increases (1~3 μm^2^/s).	[121]
	Chitosan-heparin electrostatic complexes functionalized with urease.		N/A	[122]
Triacetin	Lipase immobilized on the surface of mesoporous silica nanoparticles (MSNPs).	Self-diffusiophoresis Acetin → Acetin acid + glycerol	D_e_: 1.08 μm^2^/s in 10 mM triacetin	[124]
H^+^	Polyaniline/zinc (PANI/Zn) bilayer microtubes.	H_2_ bubble propulsion Zn + 2H^+^ → Zn^2+^ + H_2_ ↑	500 μm/s in 1.0 M HCl	[126]
	The spherical magnesium core is asymmetrically coated with TiO_2_.	H_2_ bubble propulsion Mg + 2H^+^ → Mg^2+^ + H_2_ ↑	120 μm/s in simulated gastric fluid (pH ~1.3)	[40]
	Janus CaCO_3_-SiO_2_	Self-diffusiophoresis CaCO_3_ + 2H^+^ → Ca^2+^ + CO_2_ + H_2_O	3.2 μm/s (pH 4.0)	[127]
	Hollow polystyrene loaded with CaO_2_ NPs	O_2_ bubble propulsion CaO_2_ + 4H^+^ →Ca^2+^ + 2H_2_O+ O_2_ ↑	N/A	[128]

N/A: not applicable.

**Table 2:** Comparison of composition and propulsion mechanism of physically driven MNMs.

Physical Fields	Structure and Components	Propulsion Mechanisms	Speed (μm/s) or Diffusion Coefficient (D_e_, μm^2^/s)	Ref.
Light-driven	Semiconductor polymer (SP) core encapsulated within a metal-phenolic network (MPN) shell.	NIR-II 1064 nm-triggered self-thermophoresis.	4.4 μm/s with 1.0 W/cm^2^	[146]
	Polystyrene (PS) core encapsulated within a polydopamine (PDA) shell, with indocyanine green (ICG) anchored on the surface.	NIR-I 808 nm-triggered self-thermophoresis.	N/A	[147]
	Janus double-sphere structure of mesoporous silica (MSN) and polydopamine (PDA).	NIR-I 808 nm triggered self-thermophoresis and NO bubble propulsion.	3.9 ± 0.6 μm/s with 1.5 W/cm^2^	[148]
	Bowl stomatocytes formed by block copolymerization of EG-PDLLA and deposited Au NPs on the surface.	660 nm-triggered self-thermophoresis.	124.7 ± 6.6 μm/s with 1.5 W	[149]
	Mesoporous–macroporous silica/Pt nanomotor (MMS/Pt).	NIR-triggered self-thermophoresis.	N/A	[50]
Magnetic-driven	Superparamagnetic Fe_3_O_4_ nanoparticles (NPs) were immobilized on the surface of *Spirulina platensis*.	The magnetic field drives *Spirulina platensis* to rotate around its long axis, converting the magnetic field energy into mechanical energy.	78.3 μm/s, 20 Hz, Ms 8.49 emu/g	[159]
	Ultra-small iron oxide nanoparticles (IONPs) functionalized with PEG.	Directional motion driven by a magnetic field.	100 μm/s	[34]
Ultrasound-driven	Gambogic acid (GA) and perfluorobromooctane (PFOB) encapsulated in a polylactic acid-glycolic acid copolymer (PLGA) matrix.	Acoustic interaction forces.	N/A	[160]
	Core–shell structure: Mesoporous silica nanoparticles (MSNs) are coated with mechanoluminescent SrAl_2_O_4_:Eu^2+^ (SAOE) and NIR-emitting ZnGa_2_O_4_:Cr^3+^ (ZGC).	Ultrasound triggered self-thermophoresis and NO gas propulsion.	The self-thermophoresis speed is 5.5–7.4 μm/s	[142]

N/A: not applicable.

**Table 3:** Motion mechanism and biofunction of biohybrid MNMs.

Motor Type	Composition	Propulsion Mechanisms	Functionality	Ref.
Bacteria biohybrid-propelled MNMs	*Bifidobacterium infantis*/MOF hybrids.	Anaerobic nature of *Bifidobacterium infantis*.	N/A	[165]
	*Escherichia coli* Nissle 1917@ AIEgen (INX-2).	Facultative anaerobic nature of *Escherichia coli* Nissle 1917.	NIR-I imaging, NIR-II imaging, photothermal imaging and photoacoustic imaging	[166]
Algae biohybrid-propelled MNMs	*Chlamydomonas reinhardtii* load DEPE-PEG-ADA liposome.	Phototaxis of *Chlamydomonas reinhardtii.*	Producing O_2_ while simultaneously enabling PDT for tumor treatment.	[167]
	*Chlamydomonas reinhardtii* encapsulated with pH-responsive and degradable capsule.	Autonomous motion of *Chlamydomonas reinhardtii*.	Active drug delivery in the gastrointestinal.	[171]

N/A: not applicable.

**Table 4:** MNMs for cancer therapy.

Composition	Motor Type	Tumor Model	Combined Therapeutic Means	Diagnostics	Ref.
HTiPC: Janus TiO_2_ core- mesoporous organosilica (PMO) rods modified with natural CAT.	H_2_O_2_-dependent MNMs	CT26 cells tumor bearing BALB/c mice.	PDT	N/A	[91]
JAuNR-Pt: Janus Au nanorod-Pt nanomotor JAuNR-Pt.		MCF7 cells tumor bearing BALB/c nude mice.	Release of cytotoxic Pt^2+^ ions to cause DNA damage and cell apoptosis.	NIR-II PAI	[41]
Au@MnO_2_ nanomotor: Janus Au/MnO_2_ NPs.		B16 tumor-bearing C57BL6 mice	GT	N/A	[79]
NM-si: Cationic Au nanoclusters adsorb GOx and CAT (CAuNCs@HA).	Glucose-dependent MNMs	4T1 cells tumor bearing BALB/c mice	Starvation therapy; CT	N/A	[96]
GC6@cPt ZIFs: Cisplatin (cPt) prodrug forms ZIF and encapsulates GOx and CAT.		4T1 cells tumor bearing BALB/c mice	CT; PDT; Starvation therapy	N/A	[97]
GOx@Fn proteomotors: Nanoparticles formed by cross-linking of GOx and ferritin.		4T1 cells tumor bearing BALB/c mice	CDT	N/A	[98]
a-HoMS-Au/Pt: Anisotropic hollow multishell structures incorporating Au/Pt nanoparticles.		4T1 cells tumor bearing BALB/c mice	CT	N/A	[99]
HFCA/DTX/aPD1: Heparin/folic acid binds to arginine by electrostatic interaction to form composite nanoparticles.	Arginine-dependent MNMs	B16F10 cells tumor bearing C57BL/6 mice model; MKN45 cells tumor bearing BALB/c nude mice	100 μm/s	N/A	[110]
LA-Ce6-NGs: The poly(N-vinylcaprolactam)-based nanogel co-loads arginine through hydrophobic/electrostatic interactions.		4T1 cells tumor bearing BALB/c mice	N/A	N/A	[109]
Ang-PAMSe/TLND: Polymerization of an arginine derivative (Arg-Me) and a diselenide crosslinker, modified with angiopep-2 and lonidamine.		Glioblastoma model in C57BL/6J mice	IT; CT	N/A	[108]
STING@nanomotor: Chitosan-heparin electrostatic complexes functionalized with urease and modified by the electrostatic adsorption of a STING agonist.	Urea-dependent MNMs	MB49 cells tumor bearing C57BL/6J mice	IT	N/A	[122]
CaO_2_/DOX NSs: Hollow polystyrene loaded with CaO_2_ NPs and modified AS1411 and IR-1061.	H^+^-dependent MNMs	4T1 cells tumor bearing BALB/c mice	CT; Ion interference therapy	NIR-II FLI	[128]
Chromatophore nanorobot: The FOF1-ATPase motor is embedded in lipid vesicles.		T-29 cells tumor bearing BALB/c nude mice	CT	N/A	[177]
SP@GFP nanomotors:Semiconductor polymer core encapsulated within a metal-phenolic network shell.	Light-driven MNMs	4T1 cells tumor bearing BALB/c mice	CT; PTT	N/A	[146]
PS@PDA-ICG: Polystyrene core encapsulated within a polydopamine shell, with indocyanine green anchored on the surface.		A875 cells tumor bearing BALB/c mice	PTT	NIR-II FLI	[147]
CMPCB: Janus double-sphere structure of mesoporous silica and PDA.	Light-driven MNMs	CT26 cells tumor bearing BALB/c mice	CT	N/A	[148]
BNPD-Ce6@Plt: Chlorin e6 (Ce6) was loaded onto boron nitride nanoparticles (BNPD), resulting in the formation of BNPD-Ce6-loaded lipid vesicles.		GL261 cells tumor bearing BALB/c nude mice	PDT	N/A	[180]
MMS/Pt/DOX/HF: Mesoporous–macroporous/Pt nanomotor loaded with DOX and HF.		MCF-7 cells tumor bearing BALB/c mice	CT; PTT	N/A	[50]
POMotors: Conjugating peroxidase-like P_2_W_18_Fe_4_ POMs with PDA.		S180 tumor bearing Kunming mice	PTT; CDT	N/A	[188]
4T1-JPGSs-IND: Janus Pt-Au nanospheres with 4T1 cell membrane.		4T1 cells tumor bearing BALB/c mice	PTT	PAI	[191]
ZnO_2_@PDA-Fe: ZnO_2_ modificated with PDA and Fe^2+^.		4T1 cells tumor bearing BALB/c mice	CDT; PDT	N/A	[201]
Ce/Au-Stomatocytes: Integrating Au NPs and CeO_2-x_ NPs into artificial stomatocytes.		4T1 cells tumor bearing BALB/c mice	PTT	N/A	[202]
Janus AIE/Au nanomotors: Combining AIE polymersomes with asymmetric Au nanoshells.		HeLa cells	PDT; PTT	N/A	[203]
NOSH@PEG-HCuSNPs: Hollow mesoporous CuS nanoparticles loaded with a dual gas donor.		4T1 cells tumor bearing BALB/c mice	IT; PTT; GT	PAI	[218]
APIJNS: Au_2_Pt@PMO@ICG.		HeLa tumor-bearing BALB/c mice	CDT; PTT; PDT	N/A	[219]
UMSTCA3: UCNPs@mSiO_2_-TAPP/Catalase@Au-3-MPBA		4T1 cells tumor bearing BALB/c mice	PDT; PTT	PAI; FLI	[145]
JMS NPs: Asymmetrically depositing a Au layer onto Gd-doped mesoporous silica nanoparticles.		4T1 cells tumor bearing BALB/c mice	Enhanced MRI of tumor tissues in vivo	MRI	[227]
MSP: Superparamagnetic Fe_3_O_4_ nanoparticles were immobilized on the surface of *Spirulina platensis*.	Magnetic-driven MNMs	4T1 cells tumor bearing BALB/c mice	PDT	FLI and PAI; MRI	[159]
DOX@PEG-IONPs: Ultra-small iron oxide nanoparticles functionalized with PEG and DOX.		4T1 cells tumor bearing BALB/c mice.	CT	MRI	[34]
The spherical *Chlorella pyrenoidosa* cells modified with Fe_3_O_4_ nanoparticles.		HeLa cells	CT	N/A	[157]
NP-G/P: Gambogic acid and perfluorobromooctane encapsulated in a polylactic acid-glycolic acid copolymer matrix.	Ultrasound-driven MNMs	4T1 cells tumor bearing BALB/c mice	CT; IT	N/A	[160]
APNBs: AuPt Bowl		97H cells tumor bearing BALB/c nude mice	SDT	PAI; US imaging	[204]
mSZ@PDA-NO: Core–shell structure: Mesoporous silica nanoparticles (MSNs) are coated with mechanoluminescent SrAl_2_O_4_:Eu^2+^ and NIR-emitting ZnGa_2_O_4_:Cr^3+^.	Ultrasound-driven MNMs; NO bubble propulsion.	H22 tumor-bearing Kunming mice	PTT; GT	N/A	[142]
Au NR-mSiO_2_/AIPH: The Janus Au nanorod-mesoporous silica shell is loaded with AIPH.	Ultrasound-driven MNMs; N_2_ bubble propulsion.	MCF-7 cells tumor bearing BALB/c nude mice	SDT; GT	PAI; USI	[181]
MCDP@Bif: *Bifidobacterium infantis*/MOF hybrids.	Bacteria biohybrid-propelled MNMs	4T1 cells tumor bearing BALB/c mice	CDT;	N/A	[165]
EcN@INX-2: *Escherichia coli* Nissle 1917@ AIEgen (INX-2).		CT26 cells tumor bearing BALB/c mice	PTT; PDT; IT	NIR-I FLI; NIR-II FLI; PAI; Photothermal imaging	[166]
R- Motor: *Chlamydomonas reinhardtii* load DEPE-PEG-ADA liposome.	Algae biohybrid-propelled MNMs	4T1 cells tumor bearing BALB/c mice	PDT	N/A	[167]

N/A: not applicable.

## Data Availability

No new data were created or analyzed in this literature review. Data sharing is not applicable to this article.
